# Variable-Structure Near-Space Vehicles with Time-Varying State Constraints Attitude Control Based on Switched Nonlinear System

**DOI:** 10.3390/s20030848

**Published:** 2020-02-05

**Authors:** Cong Feng, Qing Wang, Chen Liu, Changhua Hu, Xiaohui Liang

**Affiliations:** 1 Department of Automation Science and Electrical Engineering, Beihang University, Beijing 100191, China; wangqing@buaa.edu.cn (Q.W.); liangxiaohui@buaa.edu.cn (X.L.); 2 Science and Technology on Special System Simulation Laboratory, Beijing Simulation Center, Beijing 100854, China; buaalc@buaa.edu.cn; 3 Department of Automation, High-Tech Institute of Xi’an, Xi’an 710000, China; hch66619@163.com

**Keywords:** time-varying state constraints, active disturbance rejection control, variable structure near space vehicle, dynamic surface control, switched nonlinear system

## Abstract

This study is concerned with the attitude control problem of variable-structure near-space vehicles (VSNSVs) with time-varying state constraints based on switched nonlinear system. The full states of vehicles are constrained in the bounded sets with asymmetric time-varying boundaries. Firstly, considering modeling uncertainties and external disturbances, an extended state observer (ESO), including two distinct linear regions, is proposed with the advantage of avoiding the peaking value problem. The disturbance observer is utilized to estimate the total disturbances of the attitude angle and angular rate subsystems, which are described in switched nonlinear systems. Then, based on the estimation values, the asymmetric time-varying barrier Lyapunov function (BLF) is employed to construct the active disturbance rejection controller, which can ensure the full state constraints are not violated. Furthermore, to resolve the ‘explosion of complexity’ problem in backstepping control, a modified dynamic surface control is proposed. Rigorous stability analysis is given to prove that all signals of the closed-loop system are bounded. Numerical simulations are carried out to demonstrate the effectiveness of the proposed control scheme.

## 1. Introduction

The near-space vehicle (NSV) is one type of novel aerospace vehicle, which cannot only make a supersonic cruise in the atmosphere, but also perform multiple missions outside the atmosphere. Due to their superior abilities in space transportation and global strike, NSVs have been widely used in the civilian and military fields [1,2]. In comparison to the existing traditional aircrafts, NSVs have unique characteristics, such as multipurpose, multiple working modes, high mobility, large flight envelope, etc. [3] However, in near space, NSVs suffer from strong nonlinearity, serious multivariate coupling and uncertainties. The particular aerodynamic characteristics and working environment not only bring benefits, but also bring difficulties to controller design [4]. Many different approaches have been developed in the past few years. Based on the Takagi-Sugeno fuzzy models, an adaptive fault-tolerant control method was put forward for the attitude tracking of NSVs [5]. By combining the constrained control method and radial basis function neural networks, a new adaptive backstepping controller was proposed for NSVs with parametric uncertainties, external disturbances and input nonlinearities [6]. To further adapt to the large flight envelope and various task modes, variable-structure near-space vehicles (VSNSVs) were proposed which adopt variable sweep wings and retractable canard wings [7]. With the improvement in flight performance due to configuration transformation, the challenge of controller design further increases.

It should be noted that the parameters of VSNSVs (including moment of inertia, center of mass, etc.) vary seriously as the structure transforms. The above adaptive control schemes can resolve the parameter uncertainties to some extent, but the characteristics of variable structure are not fully exploited. Utilizing only one mathematical model to describe the motion of VSNSVs cannot reflect the aerodynamic characteristics comprehensively and may bring conservatism to the control design. Therefore, the switched system is introduced to model configuration transformation. The configuration transformation can be regarded as the switching of subsystems. Furthermore, the problem of control synthesis can be addressed on the basis of the switched system. In recent years, fruitful research results have been put forward to handle the controller design problem of morphing aircrafts utilizing switching control [8,9,10]. Zhang et al. [11] proposed a controller based on a switching linear parameter-varying framework for the tracking problem of flexible hypersonic vehicles. Jiang et al. [12] investigated a smooth switching linear parameter-varying control method. The parameter set can be divided automatically by a novel set partition method. Cheng et al. [13] presented a non-fragile linear parameter-varying control scheme for morphing aircraft subject to asynchronous switching and missing data. However, most of the existing literature about switching control for morphing aircrafts conduct simplification in the model by linearization, which means the model parameters can be acquired accurately and the nonlinear dynamics are underutilized. To tackle this problem, we adopt the switched nonlinear system to model the VSNSV dynamics. Even though switched nonlinear system has been generally researched in the last few years [14,15,16], the achievements for VSNSV control are rare and are yet to be further developed.

Moreover, as a practical physical control system, actuator saturation, safety specifications and command tracking performance are ubiquitous. Hence, state constraints which may lead to control effect degradation, and even instability and actuator faults, should be given attention [17]. There have been various approaches to deal with state constraints, such as model predictive control [18], reference governors [19], etc. One of various efficient approaches is the barrier Lyapunov function (BLF)-based control scheme, in which the value of the function approximates infinity when its arguments approach the constraints [20,21]. Yu [22] proposed a novel adaptive output feedback control for nonlinear systems with constant state constraints by utilizing command-filtered backstepping and state observer. Xu [23] used a method based on a combination of BLF, adaptive allocation law, and composite learning for hypersonic flight vehicles with an angle of attack constraint. Liu [24] presented reinforcement learning control to address prescribed tracking performances for hypersonic vehicles in the presence of external disturbances and heterogeneous uncertainties. However, the state constraints considered in the aforementioned literature are restricted by constant compact sets. Variable-structure near-space vehicles can make a cruise in a large flight envelope, and therefore the state constraints cannot always be kept constant. Time-varying state constraints can be more suitable for practical flight situations. To the best knowledge of the authors, existing research on time-varying state constraints for VSNSVs is rare. Liu [25] provided an adaptive control method for nonlinear systems subject to time-varying state constraints. Novel time-varying asymmetric barrier Lyapunov functions were designed in each step of backstepping to guarantee the constraints are not overstepped. However, the external disturbances were not considered in [25], and the ‘explosion of complexity’ problem due to the repeated differentiation of virtual control signals was not addressed.

In addition, considering the complicated environments in which VSNSVs work, strong wind disturbances, variations of temperature and structure deformation lead to external disturbances and parametric uncertainties which further bring difficulties in the controller design [26]. Over the past few decades, the problem of external disturbances has been investigated extensively [27]. de Jesús Rubio [28] proposed a robust linearization method for nonlinear process control, and the controller was applied to the fuel cell and manipulator. Kumar et al. [29] investigated an intelligent adaptive fractional order fuzzy sliding mode proportional integral and derivative controller for a two link robotic manipulator system. Rubio [30] put forward a structure regulator for the perturbation attenuation on the basis of the infinite structure regulator. The active disturbance rejection control (ADRC) scheme, based on extended state observer (ESO), can be an effective way to weaken the influence of external disturbances and modeling uncertainties. The essential philosophy of ADRC is to regard internal dynamics and external disturbances as extended states and estimate them utilizing an observer, then compensate it in controller design [31,32,33]. The ADRC scheme has a wide range of applications in many fields, such as hypersonic reentry vehicles [34], forced Duffing mechanical systems [35], inverter systems [36], permanent magnet synchronous motors [37], etc. Beltran-Carbajal et al. [38] put forward an output feedback control for a linear mass-spring-damper mechanical system, and an asymptotic estimation method was proposed to estimate the velocity, acceleration and disturbance signals in order to reduce the number of sensors. In [39], a novel output feedback control based on a generalized proportional integral observer for stabilization and robust tracking control of a nonlinear magnetic suspension system was investigated. Wang et al. [40] proposed a motion synchronization control technique based on linear extended state observer to handle the force fighting problem in hybrid actuation system. Zhao and Guo [41] developed an ESO-based output feedback controller for multi-input multi-output systems with mismatched uncertainty. Ran et al. [42] expanded ADRC to uncertain nonlinear systems with input time-delay based on a novel ESO. Nevertheless, there is little extant literature on the application of ADRC technology in switched nonlinear systems, and ADRC combined with time-varying asymmetric BLF also brings challenge to controller design. Motivated by the facts stated above, we consider the time-varying asymmetric BLF and active disturbance rejection control technology for VSNSV attitude tracking, in order to tackle disturbances and time-varying state constraints simultaneously.

In comparison to the current study achievements, the main contributions of this paper can be summarized as listed below.

(1) An ESO is designed to derive the accurate estimation of total disturbances for the attitude angle and angular rate subsystems. The ESO possesses two distinct linear regions to reduce the effect of peaking value problem.

(2) Time-varying asymmetric BLF is utilized to guarantee that the states of VSNSVs always remain in the time-varying constrained sets.

(3) The attitude motion of VSNSVs is modeled in the form of switched nonlinear system and the backstepping method is applied. To avoid the inherent problem of the ‘explosion of complexity’, a modified dynamic surface controller is developed. The proposed control scheme has extensive applicability compared with the existing literature.

This paper is laid out as follows. The attitude motion of VSNSVs is represented in Section 2. The extended state observer for dynamics of VSNSVs is proposed in Section 3. The controller on the basis of time-varying asymmetric barrier Lyapunov functions is proposed in Section 4. The rigorous stability analysis is put forward in Section 5. The numerical simulation results are given in Section 6, followed by conclusions in Section 7.

## 2. Mathematical Model of VSNSV

**Figure 1:**
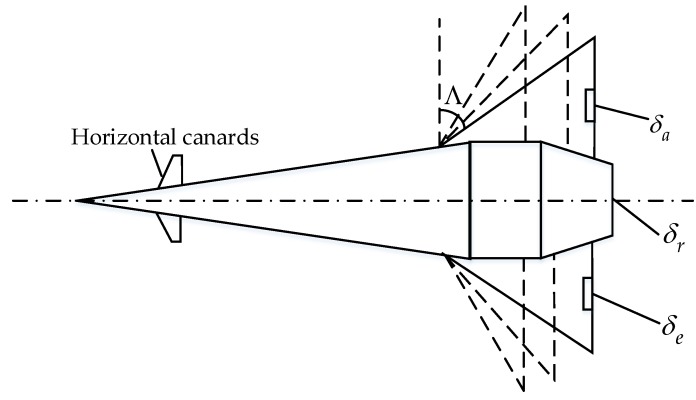
VSNSV aerodynamic model.

The VSNSV is shown as Figure 1. The maneuvering of the VSNSV is mainly executed by the engine thrust and aerodynamic control surfaces including horizontal canards, vertical tail and trailing edge elevons, which are mounted on the variable sweep wings. The horizontal canards retract at the supersonic and hypersonic speed. The sweep angle Λ can vary with different conditions of the flight. Specifically, the sweep angle keeps at $60^{\circ }$ when the vehicles carry out a supersonic flight, and the sweep angle keeps at $75^{\circ }$ during a hypersonic flight. Various structures possess different parameters, such as dynamic coefficients and the wing area. As indicated in Figure 1, the deflection angles of the left elevon, right elevon and rudder can be denoted as $\delta_{e}$, $\delta_{a}$ and $\delta_{r}$, respectively.

The attitude dynamics of the VSNSV are described in the form of switched nonlinear system as following:

(1) $$\left\{ \begin{array}{l} \dot{\Omega }=f_{a,\;\sigma (t)}^{}+g_{a}\omega +d_{a,\;\sigma (t)}^{},\\ \dot{\omega }=f_{v,\;\sigma (t)}^{}+g_{v,\;\sigma (t)}^{}M_{v}^{}+d_{v,\;\sigma (t)}^{},\\ y=\Omega , \end{array}\right.$$

where $\Omega ={\left[\alpha \;\beta \;\mu \right]}^{T}$ denotes the attitude angle vector, including the angle of attack α, the sideslip β, and the bank angle μ. $\omega ={\left[p\;q\;r\right]}^{T}$ denotes the angular rate vector, including the roll rate p, the pitch rate q, and the yaw rate r. $d_{a,\;\sigma (t)}^{}$ and $d_{v,\;\sigma (t)}^{}$ denote the total disturbances, which include modeling uncertainties and external disturbances. σ(t): [0, +∞)→Ξ={1, 2, …, n} denotes the switching signal, which is determined by sweep angle Λ. Ξ is the set of switching signals composed by right-continuous piecewise constant functions. Each value in Ξ represents a stage in which the sweep angle takes a constant value. $M_{v}^{}$ denotes the control torque vector generated by control surfaces. The other variables in Equation (1) can be represented as

$$f_{a,\;\sigma (t)}^{}={\left[f_{a1,\;\sigma (t)}^{}\;f_{a2,\;\sigma (t)}^{}\;f_{a3,\;\sigma (t)}^{}\right]}^{T},$$

$$f_{a1,\;\sigma (t)}^{}=\frac{1}{mV\cos\beta }\left(-\hat{q}S_{\sigma (t)}C_{L,\;\alpha }^{\sigma (t)}+mg\cos\gamma \cos\mu -T\sin\alpha \right),$$

$$f_{a2,\;\sigma (t)}^{}=\frac{1}{mV}\left(\hat{q}S_{\sigma (t)}C_{Y,\;\beta }^{\sigma (t)}\beta \cos\beta +mg\cos\gamma \sin\mu -T\sin\beta \cos\alpha \right),$$

$$\begin{array}{l} f_{a3,\;\sigma (t)}^{}=-\frac{g}{V}\cos\gamma \cos\mu \tan\beta +\frac{1}{mV}\hat{q}S_{\sigma (t)}C_{Y,\;\beta }^{\sigma (t)}\beta \tan\gamma \cos\mu \cos\beta \\ \;+\frac{T}{mV}[\sin\alpha (\tan\gamma \sin\mu +\tan\beta )-\cos\alpha \tan\gamma \cos\mu \sin\beta ]\\ \;+\frac{1}{mV}\hat{q}S_{\sigma (t)}C_{L,\;a}^{\sigma (t)}(\tan\gamma \sin\mu +\tan\beta ), \end{array}$$

$$g_{a}=\left[\begin{array}{ccc} -\tan\beta \cos\alpha & 1 & -\tan\beta \sin\alpha \\ \;\sin\alpha & 0 & -\cos\alpha \\ \sec\beta \cos\alpha & 0 & \sec\beta \sin\alpha \end{array}\right],$$

$$f_{v,\;\sigma (t)}^{}={\left[f_{v1,\;\sigma (t)}^{}\;f_{v2,\;\sigma (t)}^{}\;f_{v3,\;\sigma (t)}^{}\right]}^{T},$$

$$f_{v1,\;\sigma (t)}^{}={\left(J_{xx,\;\sigma (t)}^{}\right)}^{-1}\left[qr(J_{yy,\;\sigma (t)}^{}-J_{zz,\;\sigma (t)}^{})-{\dot{J}}_{xx,\;\sigma (t)}^{}p\right],$$

$$f_{v2,\;\sigma (t)}^{}={\left(J_{yy,\;\sigma (t)}^{}\right)}^{-1}\left[pr(J_{zz,\;\sigma (t)}^{}-J_{xx,\;\sigma (t)}^{})-{\dot{J}}_{yy,\;\sigma (t)}^{}q\right],$$

$$f_{v3,\;\sigma (t)}^{}={\left(J_{zz,\;\sigma (t)}^{}\right)}^{-1}\left[pq(J_{xx,\;\sigma (t)}^{}-J_{yy,\;\sigma (t)}^{})-{\dot{J}}_{zz,\;\sigma (t)}^{}r\right],$$

$$g_{v,\;\sigma (t)}^{}=\mathrm{diag}\left({\left(J_{xx,\;\sigma (t)}^{}\right)}^{-1},\;{\left(J_{yy,\;\sigma (t)}^{}\right)}^{-1},\;{\left(J_{zz,\;\sigma (t)}^{}\right)}^{-1}\right),$$

where m and V denote the mass and velocity of VSNSV, respectively. $\hat{q}$, γ, and $S^{\sigma (t)}$ denote the dynamic pressure, flight-path angle, and wing area, respectively. $C_{L,\;\alpha }^{\sigma (t)}$ and $C_{Y,\;\beta }^{\sigma (t)}$ are the aerodynamic coefficients. $J_{xx,\;\sigma (t)}^{}$, $J_{yy,\;\sigma (t)}^{}$, and $J_{zz,\;\sigma (t)}^{}$ denote the roll, pitch, and yaw moments of inertia, respectively. T denotes the engine thrust. In this study, the research focus is the attitude control of VSNSVs, which is steered by the control torque $M_{v}^{}$; thus, T is assumed to be a constant value without loss of generality [10].

The control object is to steer the VSNSV to track the desired attitude trajectory $\Omega_{\mathrm{ref}}={\left[\Omega_{\mathrm{ref}1},\;\Omega_{\mathrm{ref}2},\;\Omega_{\mathrm{ref}3}\right]}^{T}$ under the condition of internal parametric uncertainties and external disturbances. Meanwhile, the state constraints are not violated. Specifically, ${\underline{h}}_{\Omega i}(t)<\Omega_{i}<{\bar{h}}_{\Omega i}(t)$, ${\underline{h}}_{\omega i}(t)<\omega_{i}<{\bar{h}}_{\omega i}(t)$, for i=1, 2, 3, where $\Omega ={\left[\Omega_{1},\;\Omega_{2},\;\Omega_{3}\right]}^{T}$, $\omega ={\left[\omega_{1},\;\omega_{2},\;\omega_{3}\right]}^{T}$, ${\underline{h}}_{\Omega i}(t):\;R_{+}\to R$, ${\bar{h}}_{\Omega i}(t):\;R_{+}\to R$, ${\underline{h}}_{\omega i}(t):\;R_{+}\to R$, and ${\bar{h}}_{\omega i}(t):\;R_{+}\to R$.

To guarantee that the control objective is achievable, the following assumptions are proposed.

**Assumption** **1.** *The desired trajectory of the attitude angle* $\Omega_{\mathrm{ref}}$ *is continuous and twice differentiable with an unknown bound* ${\bar{\Omega }}_{r}$ *such that* ${\bar{\Omega }}_{r}\geq \max\{\left\|\Omega_{\mathrm{ref}}\right\|,\;\left\|{\dot{\Omega }}_{\mathrm{ref}}\right\|,\;\left\|{\ddot{\Omega }}_{\mathrm{ref}}\right\|\}$ *. There exist functions* ${\bar{\chi }}_{i}:\;{\mathbb{R}}_{+}\to {\mathbb{R}}_{+}$ *and* ${\underline{\chi }}_{i}:\;{\mathbb{R}}_{+}\to {\mathbb{R}}_{+}$ *satisfying* ${\underline{\chi }}_{i}(t)\leq \Omega_{\mathrm{ref}i}(t)\leq {\bar{\chi }}_{i}(t)$ *,* ${\underline{\chi }}_{i}(t)>{\underline{h}}_{\Omega i}(t)$ *, and* ${\bar{\chi }}_{i}(t)<{\bar{h}}_{\Omega i}(t)$ *for* i=1, 2, 3 *.*

**Assumption** **2.** *For any* k∈Ξ *, the compound disturbances* $d_{ai,k}^{}$ *,* $d_{vi,k}^{}$ *for* i=1, 2, 3 *and their derivatives are bounded where* $d_{a,k}^{}={\left[d_{a1,k}^{},\;d_{a2,k}^{},\;d_{a3,k}^{}\right]}^{T}$ *and* $d_{v,k}^{}={\left[d_{v1,k}^{},\;d_{v2,k}^{},\;d_{v3,k}^{}\right]}^{T}$ *There exist the positive constants* $N_{1i,k}$ *,* ${\bar{N}}_{1i,k}$ *,* $N_{2i,k}$ *and* ${\bar{N}}_{2i,k}$ *satisfying that* $\left|d_{ai,k}^{}\right|\leq N_{1i,k}$ *,* $\left|{\dot{d}}_{ai,k}^{}\right|\leq {\bar{N}}_{1i,k}$ *,* $\left|d_{vi,k}^{}\right|\leq N_{2i,k}$ *, and* $\left|{\dot{d}}_{vi,k}^{}\right|\leq {\bar{N}}_{2i,k}$ *for* i=1, 2, 3 *, respectively.*

**Assumption** **3.** *There exist positive constants* ${\underline{H}}_{\Omega i}$ *,* ${\bar{H}}_{\Omega i}$ *,* ${\underline{H}}_{\omega i}$ *,* ${\bar{H}}_{\omega i}$ *,* $H_{0}$ *satisfying* ${\underline{h}}_{\Omega i}(t)\geq {\underline{H}}_{\Omega i}$ *,* ${\bar{h}}_{\Omega i}(t)\leq {\bar{H}}_{\Omega i}$ *,* ${\underline{h}}_{\omega i}(t)\geq {\underline{H}}_{\omega i}$ *,* ${\bar{h}}_{\omega i}(t)\leq {\bar{H}}_{\omega i}$ *,* $H_{0}\geq \max\left\{\left|{\dot{\bar{h}}}_{\Omega i}(t)\right|,\;\left|{\dot{\bar{h}}}_{\omega i}(t)\right|,\;\left|{\dot{\underline{h}}}_{\;\Omega i}(t)\right|,\;\left|{\dot{\underline{h}}}_{\;\omega i}(t)\right|\right\}$ *.*

**Remark** **1.** *Assumptions 1–3 are reasonable for VSNSV attitude tracking control. Assumption 1 guarantees that the trajectory tracking is achievable, and can be found in the extant literature about attitude tracking control for near-space vehicles* [2,4,11]*. The total disturbance considered in this paper is mainly composed by modeling uncertainties and external disturbances. The accurate model parameters and the approximate parameters we used to design the controller are all bounded. The accurate parameters and the approximate parameters are all determined by the flight environment and vehicle structural parameters, which can only continuously smoothly change. Therefore, the modeling uncertainties and their derivatives are bounded. On the other hand, the external disturbances are caused by complicated temperature variation, wind disturbances, etc. As a practical physical system, the external disturbances and their time derivatives are apparently bounded. Therefore, Assumption 2 is also fairly mild and common in the literature on ESO design* [40,41,42] *and near-space vehicles-related disturbance rejection control* [6,11,34]*. Assumption 3 can be found in the literature in which output or states are constrained in time-varying sets, and guarantees that the constraints can be achieved* [21,25]*.*

**Remark** **2.** *The uncertain dynamics are considered in the total disturbance-*$d_{a,\;\sigma (t)}^{}$*and*$d_{v,\;\sigma (t)}^{}$*-in this paper. Many researchers have proposed control schemes to tackle the uncertain dynamics in the attitude motion of vehicles. Adaptive fuzzy systems are introduced to approximate the unknown functionsin the flight dynamic model, and the parameters are updated online* [5,26]*. The radial basis function neural networks are proposed to estimate the combination of parametric uncertainties and external disturbances* [2,6]*. The adaptive dynamic programming or iterative learning control are adopted to carry out auxiliary control or derive more accurate model parameters through online learning* [43,44]*. In the process of the sweep angle changing, uncertainties due to uncertain vehicle structural parameters and external disturbances severely change. The above adaptive control schemes enhance the control effect through multiple iterations and online learning, making it not suitable for fast varying models. Therefore, we tackle the uncertain dynamics as part of the total disturbance and estimate it through the proposed high-gain observer. The modified observer can track the total disturbance in a short time with the help of two linear regions.*

The following lemmas are useful to establish strict proof for the theorems in this paper.

**Lemma** **1** **[25].** *For* |x|<1 *and positive integer* p *, the following inequality holds*

$$\log\frac{1}{1-x^{2p}}<\frac{x^{2p}}{1-x^{2p}}.$$

**Lemma** **2** **[21].***Consider*K:={η∈ℝ: |η|<1}⊂ℝ*and*$W:={\mathbb{R}}^{n}\times K\subset {\mathbb{R}}^{n+1}$*are open sets. And the system*

$$\dot{\varsigma }=h(t,\;\varsigma ),$$

where $\dot{\varsigma }:={\left[\gamma ,\;\eta \right]}^{T}\in W$ and $h:{\mathbb{R}}_{+}\times W\to {\mathbb{R}}^{n+1}$ is piecewise continuous in t and locally Lipschitz in ς, uniformly in t on ${\mathbb{R}}_{+}\times W$. Suppose that there exist continuously differentiable and positive definite functions $U_{1}:\;{\mathbb{R}}^{n}\times {\mathbb{R}}_{+}\to {\mathbb{R}}_{+}$ and $U_{2}:\;K\to {\mathbb{R}}_{+}$, such that

$$\nu_{1}(\left\|\gamma \right\|)\leq U_{1}(\gamma ,\;t)\leq \nu_{2}(\left\|\gamma \right\|),$$

$$U_{2}(\eta )\to \infty \;\mathrm{as}\;\left|\eta \right|\to 1,$$

where $\nu_{1}$ and $\nu_{2}$ are class $K_{\infty }$ functions. Define $U(\varsigma )=U_{1}(\gamma ,\;t)+U_{2}(\eta )$. If η(0)∈K and the following inequality is true

$$\dot{U}=\frac{\partial U}{\partial \varsigma }h\leq -\mu U+\lambda ,$$

in the set η∈K, where μ and λ are positive constants, then η(t)∈K for t∈[0, ∞).

## 3. Extended State Observer Design

In this section, an ESO for disturbances estimation is designed. The state and extended state in the ESO system are both three-dimensional vectors to guarantee that the ESO can be directly applied in the attitude angle and angular rate subsystems of VSNSVs.

Consider the nonlinear system as follows:

(2) $$\dot{x}=\varphi (x)+\theta (x)u+d,$$

where $x,\;u,\;d\in {\mathbb{R}}^{3}$, u denotes the input signal vector, d denotes the total disturbance, and $\varphi (x),\;\theta (x)\in {\mathbb{R}}^{3\times 3}$ are both matrixes of system parameters. Then, Equation (2) is in the same form as attitude angle and angular rate dynamics in Equation (1).

**Assumption** **4.** *The disturbance* d *is bounded and differentiable with constant bound such that* $\left\|d\right\|\leq \varpi_{1}$ *,* $\left\|\dot{d}\right\|\leq \varpi_{2}$ *.*

**Remark** **3.** *Assumption 4 is common in the extant literature regarding ESO* [36,37] *and near-space vehicle adaptive control* [11,32]*, and guarantees the boundedness of total disturbance and its derivative.*

Choose d as the extended state of nonlinear system, and the corresponding extended state observer is designed as

(3) $$\left\{ \begin{array}{l} \dot{\hat{x}}=\varphi (x)+\theta (x)u+\hat{d}+\lambda_{1}\left[\frac{x-\hat{x}}{\varepsilon_{1}}+\lambda_{3}(\frac{\varepsilon_{1}}{\varepsilon_{2}}-1)\cdot h(\frac{x-\hat{x}}{\varepsilon_{1}\lambda_{3}})\right],\\ \dot{\hat{d}}=\lambda_{2}\left[\frac{x-\hat{x}}{\varepsilon_{1}^{2}}+\lambda_{3}(\frac{\varepsilon_{1}}{\varepsilon_{2}^{2}}-\frac{1}{\varepsilon_{1}})\cdot h(\frac{x-\hat{x}}{\varepsilon_{1}\lambda_{3}})\right], \end{array}\right.$$

where $\hat{x}$ and $\hat{d}$ are the estimated state vectors, $\lambda_{1}$, $\lambda_{2}$, $\lambda_{3}$, $\varepsilon_{1}$, $\varepsilon_{2}$ are all positive constants to be designed, and $\varepsilon_{1}<\varepsilon_{2}\ll 1$. For any state $x={\left[x_{1},\;x_{2},\;x_{3}\right]}^{T}$, $h(x)={\left[\mathrm{sat}(x_{1}),\;\mathrm{sat}(x_{2}),\;\mathrm{sat}(x_{3})\right]}^{T}$, where sat(x) is the general definition of saturation function defined as sat(x)=sign(x)⋅min{1, |x|}.

Then, the conclusion for the presented ESO is derived as follows.

**Theorem** **1.** *Consider the nonlinear system in Equation (2), and Assumption 4 holds, if the extended state observer is designed as Equation (3), then there exist the positive constants* $\lambda_{1}$ *,* $\lambda_{2}$ *,* $\lambda_{3}$ *,* $\varepsilon_{1}$ *, and* $\varepsilon_{2}$ *which satisfy that the estimation errors* $\left\|x-\hat{x}\right\|$ *and* $\left\|d-\hat{d}\right\|$ *will converge to a desired small neighborhood of zero for* $t\in [t_{1}+t_{2},\;+\infty )$ *, where* $t_{1}$ *and* $t_{2}$ *are constants dependent on* $\varepsilon_{1}$ *and* $\varepsilon_{2}$ *.*

**Proof.**  Define the estimation errors for system state and disturbance as $\xi_{x1}=\frac{x-\hat{x}}{\varepsilon_{1}}$, $\xi_{x2}=\frac{x-\hat{x}}{\varepsilon_{2}}$, and $\xi_{d}=d-\hat{d}$, respectively, and the estimation errors vector as $\xi ={\left[\xi_{x_{1}},\;\xi_{d}\right]}^{T}$, $\bar{\xi }={\left[\xi_{x_{2}},\;\xi_{d}\right]}^{T}$. The derivatives of the $\xi_{x1}$ and $\xi_{d}$ can be written as

(4) $$\left\{ \begin{array}{l} {\dot{\xi }}_{x_{1}}=\frac{1}{\varepsilon_{1}}\left[\xi_{d}-\lambda_{1}\xi_{x_{1}}-\lambda_{1}\lambda_{3}\left(\frac{\varepsilon_{1}}{\varepsilon_{2}}-1\right)h\left(\frac{\xi_{x_{1}}}{\lambda_{3}}\right)\right],\\ {\dot{\xi }}_{d}=\frac{1}{\varepsilon_{1}}\left[-\lambda_{2}\xi_{x_{1}}-\lambda_{2}\lambda_{3}\left(\frac{\varepsilon_{1}^{2}}{\varepsilon_{2}^{2}}-1\right)h\left(\frac{\xi_{x_{1}}}{\lambda_{3}}\right)\right]+\dot{d}. \end{array}\right.$$

 □

Choose the Lyapunov function candidate as follows

(5) $$V_{1}=\frac{1}{2}\xi_{x_{1}}^{T}\Gamma_{1}\xi_{x_{1}}+\frac{1}{2}\xi_{d}^{T}\Gamma_{2}\xi_{d}-\xi_{x_{1}}^{T}\Gamma_{3}\xi_{d}+\gamma_{1}\sum_{i=1}^{3}\int_{0}^{\xi_{x_{1}}^{i}}\mathrm{sat}(\xi_{x_{1}}^{i}/\lambda_{3})d\xi_{x_{1}}^{i},$$

where $\Gamma_{1}$, $\Gamma_{2}$, and $\Gamma_{3}$ are positive diagonal matrixes, $\gamma_{1}$ is a positive constant, $\xi_{x1}={\left[\xi_{x_{1}}^{1}\;\xi_{x_{1}}^{2}\;\xi_{x_{1}}^{3}\right]}^{T}$. The Lyapunov function candidate $V_{1}$ is used to prove the boundedness of the estimation error ξ.

Considering the first three terms on the right side of Equation (5), by taking $\Gamma_{1}-\Gamma_{3}>0$ and $\Gamma_{2}-\Gamma_{3}>0$, it can be guaranteed that

(6) $$\frac{1}{2}\xi_{x_{1}}^{T}\Gamma_{1}\xi_{x_{1}}+\frac{1}{2}\xi_{d}^{T}\Gamma_{2}\xi_{d}-\xi_{x_{1}}^{T}\Gamma_{3}\xi_{d}\geq \frac{1}{2}\xi_{x_{1}}^{T}(\Gamma_{1}-\Gamma_{3})\xi_{x_{1}}+\frac{1}{2}\xi_{d}^{T}(\Gamma_{2}-\Gamma_{3})\xi_{d}>0.$$

For the integral term in Equation (5), $\mathrm{sat}(\xi_{x_{1}}^{i}/\lambda_{3})$ is an odd function of $\xi_{x_{1}}^{i}$ for i=1, 2, 3. It can be verified that $\gamma_{1}\sum_{i=1}^{3}\int_{0}^{\xi_{x_{1}}^{i}}\mathrm{sat}(\xi_{x_{1}}^{i}/\lambda_{3})d\xi_{x_{1}}^{i}\geq 0.$ Therefore, $V_{1}$ is a positive defined and reasonable Lyapunov function candidate.

The dynamic of $V_{1}$ can be computed as

(7) $$\begin{array}{lll} {\dot{V}}_{1} & = & \frac{1}{\varepsilon_{1}}(\xi_{x_{1}}^{T}\Gamma_{1}-\xi_{d}^{T}\Gamma_{3})\left[\xi_{d}-\lambda_{1}\xi_{x_{1}}-\lambda_{1}\lambda_{3}\left(\frac{\varepsilon_{1}}{\varepsilon_{2}}-1\right)h\left(\frac{\xi_{x_{1}}}{\lambda_{3}}\right)\right]\\ & & +\frac{1}{\varepsilon_{1}}(\xi_{d}^{T}\Gamma_{2}-\xi_{x_{1}}^{T}\Gamma_{3})\left[-\lambda_{2}\xi_{x_{1}}-\lambda_{2}\lambda_{3}\left(\frac{\varepsilon_{1}^{2}}{\varepsilon_{2}^{2}}-1\right)h\left(\frac{\xi_{x_{1}}}{\lambda_{3}}\right)\right]\\ & & +\frac{\gamma_{1}}{\varepsilon_{1}}\left[\xi_{d}^{T}-\lambda_{1}\xi_{x_{1}}^{T}-\lambda_{1}\lambda_{3}\left(\frac{\varepsilon_{1}}{\varepsilon_{2}}-1\right)h^{T}\left(\frac{\xi_{x_{1}}}{\lambda_{3}}\right)\right]h\left(\frac{\xi_{x_{1}}}{\lambda_{3}}\right)\\ & & +(\xi_{d}^{T}\Gamma_{2}-\xi_{x_{1}}^{T}\Gamma_{3})\dot{d}\\ & = & \frac{1}{\varepsilon_{1}}[-\xi_{x_{1}}^{T}(\lambda_{1}\Gamma_{1}-\lambda_{2}\Gamma_{3})\xi_{x_{1}}-\xi_{d}^{T}\Gamma_{3}\xi_{d}+\xi_{x_{1}}^{T}(\Gamma_{1}-\lambda_{2}\Gamma_{2}+\lambda_{1}\Gamma_{3})\xi_{d}]\\ & & +\frac{\lambda_{3}}{\varepsilon_{1}}\xi_{d}^{T}\left[\lambda_{1}\Gamma_{3}\left(\frac{\varepsilon_{1}}{\varepsilon_{2}}-1\right)-\lambda_{2}\Gamma_{2}\left(\frac{\varepsilon_{1}^{2}}{\varepsilon_{2}^{2}}-1\right)\right]h\left(\frac{\xi_{x_{1}}}{\lambda_{3}}\right)\\ & & +\frac{\lambda_{3}}{\varepsilon_{1}}\xi_{x_{1}}^{T}\left[\lambda_{2}\Gamma_{3}\left(\frac{\varepsilon_{1}^{2}}{\varepsilon_{2}^{2}}-1\right)-\lambda_{1}\Gamma_{1}\left(\frac{\varepsilon_{1}}{\varepsilon_{2}}-1\right)\right]h\left(\frac{\xi_{x_{1}}}{\lambda_{3}}\right)\\ & & +\frac{\gamma_{1}}{\varepsilon_{1}}\left[\xi_{d}^{T}-\lambda_{1}\xi_{x_{1}}^{T}-\lambda_{1}\lambda_{3}\left(\frac{\varepsilon_{1}}{\varepsilon_{2}}-1\right)h^{T}\left(\frac{\xi_{x_{1}}}{\lambda_{3}}\right)\right]h\left(\frac{\xi_{x_{1}}}{\lambda_{3}}\right)\\ & & +(\xi_{d}^{T}\Gamma_{2}-\xi_{x_{1}}^{T}\Gamma_{3})\dot{d}. \end{array}$$

Choose $\Gamma_{1}$, $\Gamma_{2}$, $\Gamma_{3}$, and $\gamma_{1}$ according to the following restrictions

(8) $$\left\{ \begin{array}{l} \Gamma_{1}-\Gamma_{3}>0,\\ \Gamma_{2}-\Gamma_{3}>0,\\ \Gamma_{1}-\lambda_{2}\Gamma_{2}+\lambda_{1}\Gamma_{3}=0_{3\times 3},\\ \gamma_{1}I_{3}-\lambda_{2}\lambda_{3}\Gamma_{2}(\varepsilon_{1}^{2}/\varepsilon_{2}^{2}-1)+\lambda_{1}\lambda_{3}\Gamma_{3}(\varepsilon_{1}/\varepsilon_{2}-1)=0_{3\times 3}. \end{array}\right.$$

Then, Equation (7) can be rewritten as

(9) $$\begin{array}{ll} {\dot{V}}_{1}= & \frac{1}{\varepsilon_{1}}[-\xi_{x_{1}}^{T}(\lambda_{1}\Gamma_{1}-\lambda_{2}\Gamma_{3})\xi_{x_{1}}-\xi_{d}^{T}\Gamma_{3}\xi_{d}]+(\xi_{d}^{T}\Gamma_{2}-\xi_{x_{1}}^{T}\Gamma_{3})\dot{d}\\ & +\frac{1}{\varepsilon_{1}}\xi_{x_{1}}^{T}\left[-\lambda_{1}\gamma_{1}I_{3}+\lambda_{2}\lambda_{3}\left(\frac{\varepsilon_{1}^{2}}{\varepsilon_{2}^{2}}-1\right)\Gamma_{3}-\lambda_{1}\lambda_{3}\left(\frac{\varepsilon_{1}}{\varepsilon_{2}}-1\right)\Gamma_{1}\right]h\left(\frac{\xi_{x_{1}}}{\lambda_{3}}\right)\\ & -\frac{\lambda_{1}\lambda_{3}\gamma_{1}}{\varepsilon_{1}}\left(\frac{\varepsilon_{1}}{\varepsilon_{2}}-1\right)h^{T}\left(\frac{\xi_{x_{1}}}{\lambda_{3}}\right)h\left(\frac{\xi_{x_{1}}}{\lambda_{3}}\right). \end{array}$$

Furthermore, select the corresponding parameters such that $\varepsilon_{1}>\varepsilon_{2}$ and $\lambda_{1}\Gamma_{1}-\lambda_{2}\Gamma_{3}>0,$ and the following holds

(10) $$-\lambda_{1}\gamma_{1}I_{3}+\lambda_{2}\lambda_{3}\left(\frac{\varepsilon_{1}^{2}}{\varepsilon_{2}^{2}}-1\right)\Gamma_{3}-\lambda_{1}\lambda_{3}\left(\frac{\varepsilon_{1}}{\varepsilon_{2}}-1\right)\Gamma_{1}<0.$$

In order to clearly express the proof process, two compact sets are defined as $\Omega_{1}=\{\xi \in {\mathbb{R}}^{6}|V_{1}(\xi )\leq N_{1}\}$ and $\Omega_{2}=\{\xi \in {\mathbb{R}}^{6}|V_{1}(\xi )\leq N_{2}\}$, where $N_{1}$ and $N_{2}$ are both positive constants, such that $\lambda_{3}\geq \underset{\xi \in \Omega_{1}}{\max}\left\{\left|\xi_{x_{1}}^{1}\right|,\;\left|\xi_{x_{1}}^{2}\right|,\;\left|\xi_{x_{1}}^{3}\right|\right\}$ and $N_{2}=\;\max\{V_{2}(\xi (0)),\;N_{1}\}$. We complete the proof by the following two steps.

**Step 1.** First, we analyze the boundedness of ξ with help of the Lyapunov function candidate $V_{1}$. If $\xi \in \Omega_{2}-\Omega_{1}$, there exists a time $t_{1}$ satisfies that $\xi \in \Omega_{1}$ for $t\geq t_{1}$. This step is divided into two cases based on different simplification modes of $h(\xi_{x_{1}}/\lambda_{3})$.

When $\left|\xi_{x_{1}}^{i}\right|\leq \lambda_{3}$, for *i* = 1, 2, 3, it can be verified that

(11) $$h(\xi_{x_{1}}/\lambda_{3})=[\mathrm{sat}(\xi_{x_{1}}^{1}/\lambda_{3}),\;\mathrm{sat}(\xi_{x_{1}}^{2}/\lambda_{3}),\;\mathrm{sat}(\xi_{x_{1}}^{3}/\lambda_{3}{)]}^{T}={\left[\xi_{x_{1}}^{1}/\lambda_{3},\;\xi_{x_{1}}^{2}/\lambda_{3},\;\xi_{x_{1}}^{3}/\lambda_{3}\right]}^{T}.$$

Combined with Assumption 4, Equations (5) and (9) can be rewritten as

(12) $$\begin{array}{l} V_{1}=\frac{1}{2}\xi_{x_{1}}^{T}\Gamma_{1}\xi_{x_{1}}+\frac{1}{2}\xi_{d}^{T}\Gamma_{2}\xi_{d}-\xi_{x_{1}}^{T}\Gamma_{3}\xi_{d}+\frac{\gamma_{1}}{2\lambda_{3}}{\left[{\left(\xi_{x_{1}}^{1}\right)}^{2},\;{\left(\xi_{x_{1}}^{2}\right)}^{2},\;{\left(\xi_{x_{1}}^{3}\right)}^{2}\right]}^{T}\\ \;\leq \frac{1}{2}\left\|\Gamma_{1}+\Gamma_{3}+\frac{\gamma_{1}}{\lambda_{3}}I_{3}\right\|{\left\|\xi_{x_{1}}\right\|}^{2}+\frac{1}{2}\left\|\Gamma_{2}+\Gamma_{3}\right\|{\left\|\xi_{d}\right\|}^{2}, \end{array}$$

(13) $$\begin{array}{l} {\dot{V}}_{1}=\frac{1}{\varepsilon_{1}}[-\xi_{x_{1}}^{T}(\lambda_{1}\Gamma_{1}-\lambda_{2}\Gamma_{3})\xi_{x_{1}}-\xi_{d}^{T}\Gamma_{3}\xi_{d}]+(\xi_{d}^{T}\Gamma_{2}-\xi_{x_{1}}^{T}\Gamma_{3})\dot{d}\\ \;+\frac{1}{\varepsilon_{1}}\xi_{x_{1}}^{T}\left[-\frac{\lambda_{1}\gamma_{1}}{\lambda_{3}}I_{3}+\lambda_{2}\Gamma_{3}\left(\frac{\varepsilon_{1}^{2}}{\varepsilon_{2}^{2}}-1\right)-\lambda_{1}\Gamma_{1}\left(\frac{\varepsilon_{1}}{\varepsilon_{2}}-1\right)\right]\xi_{x_{1}}-\frac{\lambda_{1}\gamma_{1}}{\varepsilon_{1}\lambda_{3}}\left(\frac{\varepsilon_{1}}{\varepsilon_{2}}-1\right)\xi_{x_{1}}^{T}\xi_{x_{1}}\\ \;=-\frac{1}{\varepsilon_{1}}\xi_{x_{1}}^{T}\left(\frac{\lambda_{1}\gamma_{1}}{\lambda_{3}}\frac{\varepsilon_{1}}{\varepsilon_{2}}I_{3}-\lambda_{2}\frac{\varepsilon_{1}^{2}}{\varepsilon_{2}^{2}}\Gamma_{3}+\lambda_{1}\frac{\varepsilon_{1}}{\varepsilon_{2}}\Gamma_{1}\right)\xi_{x_{1}}-\frac{1}{\varepsilon_{1}}\xi_{d}^{T}\Gamma_{3}\xi_{d}+(\xi_{d}^{T}\Gamma_{2}-\xi_{x_{1}}^{T}\Gamma_{3})\dot{d}\\ \;\leq -\frac{1}{2\varepsilon_{1}}(\xi_{x_{1}}^{T}P_{1}\xi_{x_{1}}+\xi_{d}^{T}\Gamma_{3}\xi_{d})+\left\|\xi \right\|q_{1}\varpi_{2}-\frac{1}{2\varepsilon_{1}}q_{2}{\left\|\xi \right\|}^{2}, \end{array}$$

where $P_{1}=\frac{\lambda_{1}\gamma_{1}}{\lambda_{3}}\frac{\varepsilon_{1}}{\varepsilon_{2}}I_{3}-\lambda_{2}\frac{\varepsilon_{1}^{2}}{\varepsilon_{2}^{2}}\Gamma_{3}+\lambda_{1}\frac{\varepsilon_{1}}{\varepsilon_{2}}\Gamma_{1}$, $q_{1}=\left\|\Gamma_{2}\right\|+\left\|\Gamma_{3}\right\|$, and $q_{2}=\lambda_{\min}\left(\left[\begin{array}{cc} P_{1} & \\ & \Gamma_{3} \end{array}\right]\right)$. Combined with Equation (10) and $\lambda_{1}\Gamma_{1}-\lambda_{2}\Gamma_{3}>0$, it can be guaranteed that $P_{1}>0$.

Take $\varepsilon_{1}\leq \frac{q_{2}}{2q_{1}\varpi_{2}}\underset{\xi \in \Omega_{2}-\Omega_{1}}{\min}\left\|\xi \right\|$, and it can be verified

(14) $${\dot{V}}_{1}\;\leq -\frac{1}{2\varepsilon_{1}}(\xi_{x_{1}}^{T}P_{1}\xi_{x_{1}}+\xi_{d}^{T}\Gamma_{3}\xi_{d})\leq -\frac{q_{3}}{\varepsilon_{1}}V_{1},$$

where $q_{3}=\min\left\{\lambda_{\min}(P_{1})/\left\|\Gamma_{1}+\Gamma_{3}+\frac{\gamma_{1}}{\lambda_{3}}I_{3}\right\|,\;\lambda_{\min}(\Gamma_{3})/\left\|\Gamma_{2}+\Gamma_{3}\right\|\right\}$.

When there exists a $\left|\xi_{x_{1}}^{j}\right|>\lambda_{3}$, for any j∈{1, 2, 3}, it can be verified that

(15) $$\mathrm{sat}(\xi_{x_{1}}^{j}/\lambda_{3})=\mathrm{sign}(\xi_{x_{1}}^{j})$$

For the simplicity of expression, the number of $\xi_{x_{1}}^{j}$ that satisfy $\left|\xi_{x_{1}}^{j}\right|>\lambda_{3}$ is denoted by m for j=1, 2, 3, and denote r as the number of $\xi_{x_{1}}^{i}$ that satisfy $\left|\xi_{x_{1}}^{i}\right|\leq \lambda_{3}$, for i=1, 2, 3.

Substituting Equation (15) into Equations (5) and (9) yields

(16) $$\begin{array}{l} V_{1}=\frac{1}{2}\xi_{x_{1}}^{T}\Gamma_{1}\xi_{x_{1}}+\frac{1}{2}\xi_{d}^{T}\Gamma_{2}\xi_{d}-\xi_{x_{1}}^{T}\Gamma_{3}\xi_{d}+\sum_{i=1}^{r}\frac{\gamma_{1}}{2\lambda_{3}}{\left(\xi_{x_{1}}^{i}\right)}^{2}+\gamma_{1}\sum_{j=1}^{m}\left(\left|\xi_{x_{1}}^{i}\right|-\frac{\lambda_{3}}{2}\right)\\ \;\leq \frac{1}{2}\xi_{x_{1}}^{T}(\Gamma_{1}+\Gamma_{3}+\frac{\gamma_{1}}{\lambda_{3}}I_{3})\xi_{x_{1}}+\frac{1}{2}\xi_{d}^{T}(\Gamma_{2}+\Gamma_{3})\xi_{d}+\gamma_{1}\sum_{j=1}^{m}\left(\left|\xi_{x_{1}}^{i}\right|-\frac{\lambda_{3}}{2}\right)\\ \;\leq \frac{1}{2}\left\|\Gamma_{1}+\Gamma_{3}+\frac{\gamma_{1}}{\lambda_{3}}I_{3}\right\|{\left\|\xi_{x_{1}}\right\|}^{2}+\frac{1}{2}\left\|\Gamma_{2}+\Gamma_{3}\right\|{\left\|\xi_{d}\right\|}^{2}+\gamma_{1}\sum_{j=1}^{m}\left(\left|\xi_{x_{1}}^{i}\right|-\frac{\lambda_{3}}{2}\right). \end{array}$$

(17) $$\begin{array}{lll} {\dot{V}}_{1} & \leq & \frac{1}{\varepsilon_{1}}[-\xi_{x_{1}}^{T}(\lambda_{1}\Gamma_{1}-\lambda_{2}\Gamma_{3})\xi_{x_{1}}-\xi_{d}^{T}\Gamma_{3}\xi_{d}]+\left\|\xi \right\|q_{1}\varpi_{2}\\ & & -\frac{1}{\varepsilon_{1}}\sum_{j=1}^{m}\left|\xi_{x_{1}}^{j}\right|P_{2}^{jj}-\sum_{j=1}^{m}\left[\frac{\lambda_{1}\lambda_{3}\gamma_{1}}{\varepsilon_{1}}\left(\frac{\varepsilon_{1}}{\varepsilon_{2}}-1\right)\right]\\ & \leq & -\frac{1}{2\varepsilon_{1}}\xi_{x_{1}}^{T}(\lambda_{1}\Gamma_{1}-\lambda_{2}\Gamma_{3})\xi_{x_{1}}-\frac{1}{2\varepsilon_{1}}\xi_{d}^{T}\Gamma_{3}\xi_{d}+\left\|\xi \right\|q_{1}\varpi_{2}\\ & & -\frac{1}{2\varepsilon_{1}}q_{4}{\left\|\xi \right\|}^{2}-\frac{q_{5}}{\varepsilon_{1}}\sum_{j=1}^{m}\left(\left|\xi_{x_{1}}^{j}\right|-\frac{\lambda_{3}}{2}\right), \end{array}$$

where $P_{2}=\lambda_{1}\gamma_{1}I_{3}-\lambda_{2}\lambda_{3}\Gamma_{3}\left(\frac{\varepsilon_{1}^{2}}{\varepsilon_{2}^{2}}-1\right)+\lambda_{1}\lambda_{3}\left(\frac{\varepsilon_{1}}{\varepsilon_{2}}-1\right)\Gamma_{1}>0$, $P_{2}^{jj}$ is the j-th diagonal element of the matrix $P_{2}$, $q_{4}=\lambda_{\min}\left(\left[\begin{array}{cc} \lambda_{1}\Gamma_{1}-\lambda_{2}\Gamma_{3} & \\ & \Gamma_{3} \end{array}\right]\right)$, and $q_{5}=\lambda_{\min}(P_{2})$.

Choose an appropriate $\varepsilon_{1}$ such that $\varepsilon_{1}\leq \frac{q_{4}}{2q_{1}\varpi_{2}}\underset{\xi \in \Omega_{2}-\Omega_{1}}{\min}\left\|\xi \right\|$ and we can arrive at

(18) $$\begin{array}{lll} {\dot{V}}_{1} & \leq & -\frac{1}{2\varepsilon_{1}}\xi_{x_{1}}^{T}(\lambda_{1}\Gamma_{1}-\lambda_{2}\Gamma_{3})\xi_{x_{1}}-\frac{1}{2\varepsilon_{1}}\xi_{d}^{T}\Gamma_{3}\xi_{d}-\frac{q_{5}}{\varepsilon_{1}}\sum_{j=1}^{m}\left(\left|\xi_{x_{1}}^{j}\right|-\frac{\lambda_{3}}{2}\right)\\ & \leq & -\frac{q_{6}}{\varepsilon_{1}}V_{1}, \end{array}$$

where $q_{6}=\min\left\{\lambda_{\min}(\lambda_{1}\Gamma_{1}-\lambda_{2}\Gamma_{3})/\left\|\Gamma_{1}+\Gamma_{3}+\frac{\gamma_{1}}{\lambda_{3}}I_{3}\right\|,\;\lambda_{\min}(\Gamma_{3})/\left\|\Gamma_{2}+\Gamma_{3}\right\|,\;\frac{q_{5}}{\gamma_{1}}\right\}$.

Comprehensively analyze the above two situations as shown in Equations (14) and (18), combined with the comparison principle of ordinary differential equations, it can be achieved that

(19) $$V_{1}(\xi (t))\leq e^{-\frac{q_{7}}{\varepsilon_{1}}t}V_{1}(\xi (0)),$$

where $q_{7}=\min\{q_{3},\;q_{6}\}$, and $\varepsilon_{1}\leq \min\{\frac{q_{2}}{2q_{1}v_{2}}\underset{\xi \in \Omega_{2}-\Omega_{1}}{\min}\left\|\xi \right\|,\;\frac{q_{4}}{2q_{1}\varpi_{2}}\underset{\xi \in \Omega_{2}-\Omega_{1}}{\min}\left\|\xi \right\|\}$.

From Equation (19) it can be concluded that $\xi \in \Omega_{1}$, for $t\geq t_{1}=\frac{\varepsilon_{1}}{q_{7}}\ln\left(\frac{N_{2}}{N_{1}}\right)$. This means that once $\xi \in \Omega_{2}-\Omega_{1}$, $V_{1}(\xi )$ decreases until $\xi \in \Omega_{1}$ again.

**Step 2.** In this step, we prove the main conclusion of Theorem 1. The following analysis in on the basis of $\xi \in \Omega_{1}$, which means $\left|\xi_{x_{1}}^{i}\right|\leq \lambda_{3}$, for i=1, 2, 3. The condition holds when $t\geq t_{1}$. Compute the derivatives of $\bar{\xi }$ and $\xi_{d}$ as follows

(20) $$\left\{ \begin{array}{l} {\dot{\xi }}_{x2}=\frac{1}{\varepsilon_{2}}(\xi_{d}-\lambda_{1}\xi_{x2}),\\ {\dot{\xi }}_{d}=-\frac{\lambda_{2}}{\varepsilon_{2}}\xi_{x2}+\dot{d}. \end{array}\right.$$

Choose the Lyapunov function candidate as

(21) $$V_{2}=\frac{1}{2}\xi_{x_{2}}^{T}\Gamma_{1}\xi_{x_{2}}+\frac{1}{2}\xi_{d}^{T}\Gamma_{2}\xi_{d}-\xi_{x_{2}}^{T}\Gamma_{3}\xi_{d}.$$

The Lyapunov function candidate $V_{2}$ is different from $V_{1}$ which is used to prove the boundedness of ξ.

By the help of Young’s inequality, it can be verified that

(22) $$\frac{1}{2}\xi_{x_{2}}^{T}(\Gamma_{1}-\Gamma_{3})\xi_{x_{2}}+\frac{1}{2}\xi_{d}^{T}(\Gamma_{2}-\Gamma_{3})\xi_{d}\leq V_{2}\leq \frac{1}{2}\xi_{x_{2}}^{T}(\Gamma_{1}+\Gamma_{3})\xi_{x_{2}}+\frac{1}{2}\xi_{d}^{T}(\Gamma_{2}+\Gamma_{3})\xi_{d}.$$

Considering the relation between $\Gamma_{1}$, $\Gamma_{2}$, and $\Gamma_{3}$ in Equation (8), $V_{2}$ is positive define and a reasonable Lyapunov function candidate.

Combined with Equation (8), computing the dynamics of $V_{2}$ yields

(23) $${\dot{V}}_{2}=\frac{1}{\varepsilon_{2}}\left[-\xi_{x_{2}}^{T}(\lambda_{1}\Gamma_{1}-\lambda_{2}\Gamma_{3})\xi_{x_{2}}-\xi_{d}^{T}\Gamma_{3}\xi_{d}\right]+\left(\xi_{d}^{T}\Gamma_{2}-\xi_{x_{2}}^{T}\Gamma_{3}\right)\dot{d}$$

Substitute Equation (21) into Equation (23) and we can get

(24) $$\begin{array}{l} {\dot{V}}_{2}\leq -\frac{1}{\varepsilon_{2}}\left[\xi_{x_{2}}^{T}(\lambda_{1}\Gamma_{1}-\lambda_{2}\Gamma_{3})\xi_{x_{2}}+\xi_{d}^{T}\Gamma_{3}\xi_{d}\right]+q_{1}\varpi_{2}\left\|\bar{\xi }\right\|\\ \;\leq -\frac{q_{8}}{\varepsilon_{2}}V_{2}+q_{1}\varpi_{2}\sqrt{\frac{V_{2}}{q_{9}}}, \end{array}$$

where $q_{8}=\min\left\{\frac{2\lambda_{\min}(\lambda_{1}\Gamma_{1}-\lambda_{2}\Gamma_{3})}{\left\|\Gamma_{1}+\Gamma_{3}\right\|},\;\frac{2\lambda_{\min}(\Gamma_{3})}{\left\|\Gamma_{2}+\Gamma_{3}\right\|}\right\}$, $q_{9}=\frac{1}{2}\lambda_{\min}\left(\left[\begin{array}{cc} \Gamma_{1}-\Gamma_{3} & \\ & \Gamma_{2}-\Gamma_{3} \end{array}\right]\right)$.

It can be seen that when $V_{2}$ exceeds $4\varepsilon_{2}^{2}q_{1}^{2}\varpi_{2}^{2}/q_{9}q_{8}^{2}$

(25) $$\begin{array}{l} {\dot{V}}_{2}\leq -\frac{1}{\varepsilon_{2}}\left[\xi_{x_{2}}^{T}(\lambda_{1}\Gamma_{1}-\lambda_{2}\Gamma_{3})\xi_{x_{2}}+\xi_{d}^{T}\Gamma_{3}\xi_{d}\right]+q_{1}\varpi_{2}\left\|\bar{\xi }\right\|\\ \;\leq -\frac{q_{8}}{2\varepsilon_{2}}V_{2}<0. \end{array}$$

Combined with the comparison principle of the ordinary differential equations, it can be derived

(26) $$V_{2}(\bar{\xi }(t))\leq e^{-\frac{q_{8}}{2\varepsilon_{2}}(t-t_{1})}V_{2}(\bar{\xi }(t_{1}))$$

Considering the definition of ξ and $\bar{\xi }$, we can deduce that there exists a constant $N_{3}$ that satisfies that $V_{2}(\bar{\xi }(t))\leq N_{3}$ for $t\geq t_{1}$. Then, for $t\geq t_{1}+t_{2}$, where $t_{2}=\frac{2\varepsilon_{2}}{q_{8}}\ln\left(\frac{\max\{N_{3},\;4\varepsilon_{2}^{2}q_{1}^{2}\varpi_{2}^{2}/q_{9}q_{8}^{2}\}}{4\varepsilon_{2}^{2}q_{1}^{2}\varpi_{2}^{2}/q_{9}q_{8}^{2}}\right)$, it can be achieved that

(27) $$V_{2}\leq \frac{4\varepsilon_{2}^{2}q_{1}^{2}\varpi_{2}^{2}}{q_{9}q_{8}^{2}}.$$

Furthermore, combined with Equation (22), we can arrive at

(28) $$\left\|\bar{\xi }(t)\right\|\leq \sqrt{\frac{V_{2}(\bar{\xi }(t))}{q_{9}}}\leq \frac{2\varepsilon_{2}q_{1}\varpi_{2}}{q_{8}q_{9}},$$

(29) $$\|x(t)-\hat{x}(t)\|=\varepsilon_{2}\|\xi_{x2}(t)\|\leq \varepsilon_{2}\left\|\bar{\xi }(t)\right\|\leq \frac{2\varepsilon_{2}^{2}q_{1}\varpi_{2}}{q_{8}q_{9}},$$

(30) $$\|d(t)-\hat{d}(t)\|=\|\xi_{d}(t)\|\leq \left\|\bar{\xi }(t)\right\|\leq \frac{2\varepsilon_{2}q_{1}\varpi_{2}}{q_{8}q_{9}}.$$

From Equations (29) and (30), it can be noted that $\left\|x-\hat{x}\right\|$ and $\left\|d-\hat{d}\right\|$ converge to a desired small neighborhood of zero for $t\in [t_{1}+t_{2},\;+\infty )$. The proof of Theorem 1 has been completed.

**Remark** **4.** *It should be pointed out whether* $\left|\frac{x^{i}-{\hat{x}}^{i}}{\varepsilon_{1}\lambda_{3}}\right|>1$ *determines the form of the ESO due to the saturation function* $h\left(\frac{x-\hat{x}}{\varepsilon_{1}\lambda_{3}}\right)$ *, for* i=1, 2, 3 *. When* $\left|\frac{x^{i}-{\hat{x}}^{i}}{\varepsilon_{1}\lambda_{3}}\right|\leq 1$ *, for* i=1, 2, 3 *, the corresponding ESO system becomes the following*

(31) $$\left\{ \begin{array}{l} {\dot{\hat{x}}}^{i}=\varphi^{i}(x)+\theta^{i}(x)u+{\hat{d}}^{i}+\lambda_{1}\frac{x^{i}-{\hat{x}}^{i}}{\varepsilon_{2}},\\ {\dot{\hat{d}}}^{i}=\lambda_{2}\frac{x^{i}-{\hat{x}}^{i}}{\varepsilon_{2}^{2}}. \end{array}\right.$$

Apparently, the estimation error $\left(x^{i}-{\hat{x}}^{i}\right)$ lies in the linear region of the saturation function, and the ESO has a relatively slower dynamic characteristic due to the larger parameter $\varepsilon_{2}$. When $\left|\frac{x^{i}-{\hat{x}}^{i}}{\varepsilon_{1}\lambda_{3}}\right|>1$, for i=1, 2, 3, the ESO dynamics have the following form

$$\left\{ \begin{array}{l} {\dot{\hat{x}}}^{i}=\varphi^{i}(x)+\theta^{i}(x)u+{\hat{d}}^{i}+\lambda_{1}\left[\frac{x^{i}-{\hat{x}}^{i}}{\varepsilon_{1}}+\lambda_{3}\left(\frac{\varepsilon_{1}}{\varepsilon_{2}}-1\right)\cdot \mathrm{sign}(x^{i}-{\hat{x}}^{i})\right],\\ {\dot{\hat{d}}}^{i}=\lambda_{2}\left[\frac{x^{i}-{\hat{x}}^{i}}{\varepsilon_{1}^{2}}+\lambda_{3}\left(\frac{\varepsilon_{1}}{\varepsilon_{2}^{2}}-\frac{1}{\varepsilon_{1}}\right)\cdot \mathrm{sign}(x^{i}-{\hat{x}}^{i})\right]. \end{array}\right.$$

It can be seen that the smaller parameter $\varepsilon_{1}$ guarantees that the ESO system works in higher linear gain. In most of the existing literature about disturbance observers, there exists a tradeoff between the fast reconstruction of the states and the steady-state error [40,41,42]. High gain disturbance observers can attenuate the steady-state estimation error due to the modeling uncertainty, but increasing the gain leads to a higher sensitivity to measurement noise. The tradeoff seriously limits the performance of the disturbance observer. From the above analysis, we can see that when the estimation error is big as $\left|(x^{i}-{\hat{x}}^{i})/\varepsilon_{1}\lambda_{3}\right|>1$, the observer works in the region of the larger gain for fast state reconstruction, and when the estimation error is small as $\left|(x^{i}-{\hat{x}}^{i})/\varepsilon_{1}\lambda_{3}\right|\leq 1$, the observer forces a smaller gain to reduce the effect of noise. The sliding mode-like terms (the terms with sign(⋅)) are introduced to improve the effect of the extended state observer [32].

**Remark** **5.** *Considering the ESO out of saturation in Equation (31), rewrite the derivatives of estimation errors as follows*

(32) $$\dot{\bar{\xi }}=\frac{1}{\varepsilon_{2}}\Psi \bar{\xi }+{\left[0_{1\times 3}\;\dot{d}\right]}^{T},$$

where $\Psi =\left[\begin{array}{cc} -\lambda_{1} & 1\\ -\lambda_{2} & 0 \end{array}\right]$. The matrix form in Equation (32) is similar to the high-gain observer in [33,37]. Therefore, the design method of parameters can refer to the existing literature [33,37]. Based on pole assignment, the specific selection principle is to make the eigenvalues of Ψ have negative real parts with modest absolute values. For example, a common solution is $\lambda_{1}=2\omega$ and $\lambda_{2}=\omega^{2}$, where ω is a positive constant, and the eigenvalues of Ψ are both −ω. In addition, from Equation (29) and Equation (30), $\varepsilon_{2}$ should be chosen far less than 1 with the purpose of achieving smaller estimation errors bounds. $\varepsilon_{1}$ should be set that $\varepsilon_{1}<\varepsilon_{2}\ll 1$ to guarantee the different observer characteristics in two linear regions, as described in Remark 4.

## 4. Controller Design

On the basis of the multiple-time-scale characteristics of VSNSVs [10], the angular rate system has faster dynamic performance, which is called fast-loop, and correspondingly the attitude angle system is the slow-loop. Hence, in this section, we design controllers for attitude angle loop and angular rate loop, respectively. The ESO described in Equation (3) is introduced to estimate the total disturbance in each loop. In this paper, the change of sweep angle Λ is time-driven, and the switching signals σ(t) are therefore independent parameters as in [10]. For any k∈Ξ, the sweep angle Λ remains at a specific value, and the control law is designed for each subsystem along the backstepping control scheme.

### 4.1. Control Law Design for Attitude Angle System

Considering the first equation in the VSNSV dynamics (1), regard $d_{a}^{k}$ as the extended state, and the ESO designed in Equation (3) is introduced as follows to estimate the total disturbances $d_{a}^{k}$.

(33) $$\left\{ \begin{array}{l} \dot{\hat{\Omega }}=f_{a,k}^{}+g_{a}\omega +{\hat{d}}_{a,k}^{}+\lambda_{1\Omega ,\;k}\left[\frac{\Omega -\hat{\Omega }}{\varepsilon_{1\Omega ,\;k}}+\lambda_{3\Omega ,\;k}(\frac{\varepsilon_{1\Omega ,\;k}}{\varepsilon_{2\Omega ,\;k}}-1)\cdot h(\frac{\Omega -\hat{\Omega }}{\varepsilon_{1\Omega ,\;k}\lambda_{3\Omega ,\;k}})\right],\\ {\dot{\hat{d}}}_{a,k}^{}=\lambda_{2\Omega ,\;k}\left[\frac{\Omega -\hat{\Omega }}{\varepsilon_{1\Omega ,\;k}^{2}}+\lambda_{3\Omega ,\;k}(\frac{\varepsilon_{1\Omega ,\;k}}{\varepsilon_{2\Omega ,\;k}^{2}}-\frac{1}{\varepsilon_{1\Omega ,\;k}})\cdot h(\frac{\Omega -\hat{\Omega }}{\varepsilon_{1\Omega ,\;k}\lambda_{3\Omega ,\;k}})\right], \end{array}\right.$$

where $\lambda_{1\Omega ,k}$, $\lambda_{2\Omega ,k}$, $\lambda_{3\Omega ,\;k}$, $\varepsilon_{1\Omega ,\;k}$, and $\varepsilon_{2\Omega ,\;k}$ are positive constants to be selected and $\varepsilon_{1\Omega ,\;k}<\varepsilon_{2\Omega ,\;k}\ll 1$.

According to the Theorem 1 and Assumption 2, it can be guaranteed that there exist $\lambda_{1\Omega ,k}$, $\lambda_{2\Omega ,k}$, $\lambda_{3\Omega ,\;k}$, $\varepsilon_{1\Omega ,\;k}$, and $\varepsilon_{2\Omega ,\;k}$ such that the estimation errors $\left\|\Omega -\hat{\Omega }\right\|$ and $\left\|d_{a,k}^{}-{\hat{d}}_{a,k}^{}\right\|$ will converge to a desired small neighborhood of zero for $t\in [t_{\Omega ,\;k},\;+\infty )$, where $t_{\Omega }$ is a positive constant related to $\varepsilon_{1\Omega ,\;k}$ and $\varepsilon_{2\Omega ,\;k}$. In particular, we suppose $\left\|{\tilde{d}}_{a,k}^{}\right\|=\left\|d_{a,k}^{}-{\hat{d}}_{a,k}^{}\right\|\leq D_{a,\;k}$ for $t\in [t_{\Omega ,\;k},\;+\infty )$.

Define the attitude angle tracking error as $e_{\Omega i}=\Omega_{i}-\Omega_{\mathrm{ref}i}$ for i=1, 2, 3, and the derivate of $e_{\Omega i}$ is

(34) $${\dot{e}}_{\Omega i}=f_{ai,k}^{}+{\left(g_{a}\omega \right)}_{i}+d_{ai,k}^{}-{\dot{\Omega }}_{\mathrm{ref}i}.$$

Consider the time-varying asymmetric BLF candidate as

(35) $$V_{\Omega }=\sum_{i=1}^{3}\frac{o(e_{\Omega i})}{2p}\log\frac{{\bar{r}}_{\;\Omega i}^{2p}}{{\bar{r}}_{\;\Omega i}^{2p}-e_{\Omega i}^{2p}}+\frac{1-o(e_{\Omega i})}{2p}\log\frac{{\underline{r}}_{\;\Omega i}^{2p}}{{\underline{r}}_{\;\Omega i}^{2p}-e_{\Omega i}^{2p}},$$

where ${\bar{r}}_{\;\Omega i}^{}(t)={\bar{h}}_{\Omega i}(t)-\Omega_{\mathrm{ref}i}(t)$, ${\underline{r}}_{\;\Omega i}^{}(t)=\Omega_{\mathrm{ref}i}(t)-{\underline{h}}_{\Omega i}(t)$, p is a positive integer and

$$o(x)=\{\begin{array}{c} 1,\;x>0,\\ 0,\;x\leq 0. \end{array}$$

On the basis of the definitions of ${\bar{r}}_{\;\Omega i}^{}(t)$ and ${\underline{r}}_{\;\Omega i}^{}(t)$, and utilizing Assumptions 1 and 3, it can be verified that ${\bar{R}}_{1\Omega i}^{}\leq {\bar{r}}_{\;\Omega i}^{}(t)\leq {\bar{R}}_{2\Omega i}^{}$, ${\underline{R}}_{\;1\Omega i}^{}\leq {\underline{r}}_{\;\Omega i}^{}(t)\leq {\underline{R}}_{\;2\Omega i}^{}$, where ${\bar{R}}_{1\Omega i}^{}$, ${\bar{R}}_{2\Omega i}^{}$, ${\underline{R}}_{\;1\Omega i}^{}$, and ${\underline{R}}_{\;2\Omega i}^{}$ are all constants.

For the convenience of expression, we make change of coordinate as

(36) $$\left\{ \begin{array}{l} {\bar{\eta }}_{\Omega i}^{}=\frac{e_{\Omega i}^{}}{{\bar{r}}_{\;\Omega i}^{}},\;{\underline{\eta }}_{\Omega i}^{}=\frac{e_{\Omega i}^{}}{{\underline{r}}_{\;\Omega i}^{}},\\ \eta_{\Omega i}=o(e_{\Omega i}){\bar{\eta }}_{\Omega i}^{}+(1-o(e_{\Omega i})){\underline{\eta }}_{\Omega i}^{}. \end{array}\right.$$

Then, Equation (35) can be rewritten as

(37) $$V_{\Omega }=\sum_{i=1}^{3}\frac{1}{2p}\log\frac{1}{1-\eta_{\;\Omega i}^{2p}}.$$

Apparently, under the premise of $\left|\eta_{\Omega i}\right|<1$, $V_{\Omega }$ is positive, definite and continuously differentiable. Combined with Equation (34), the dynamics of $V_{\Omega }$ is

(38) $$\begin{array}{lll} {\dot{V}}_{\Omega } & = & \sum_{i=1}^{3}\left[\frac{o(e_{\Omega i}){\bar{\eta }}_{\;\Omega i}^{2p-1}}{{\bar{r}}_{\;\Omega i}(1-{\bar{\eta }}_{\;\Omega i}^{2p})}\left({\dot{e}}_{\Omega i}-e_{\Omega i}\frac{{\dot{\bar{r}}}_{\;\Omega i}}{{\bar{r}}_{\;\Omega i}}\right)+\frac{(1-o(e_{\Omega i})){\underline{\eta }}_{\;\Omega i}^{2p-1}}{{\underline{r}}_{\;\Omega i}(1-{\underline{\eta }}_{\;\Omega i}^{2p})}\left({\dot{e}}_{\Omega i}-e_{\Omega i}\frac{{\dot{\underline{r}}}_{\;\Omega i}}{{\underline{r}}_{\;\Omega i}}\right)\right]\\ & = & \sum_{i=1}^{3}[\frac{\eta_{\;\Omega i}^{2p}}{e_{\Omega i}(1-\eta_{\;\Omega i}^{2p})}\left(f_{ai,k}^{}+{\left(g_{a}\omega \right)}_{i}+d_{ai,k}^{}-{\dot{\Omega }}_{refi}\right)\\ & & -\frac{o(e_{\Omega i}){\bar{\eta }}_{\;\Omega i}^{2p-1}}{{\bar{r}}_{\;\Omega i}(1-{\bar{\eta }}_{\;\Omega i}^{2p})}e_{\Omega i}\frac{{\dot{\bar{r}}}_{\;\Omega i}}{{\bar{r}}_{\;\Omega i}}-\frac{(1-o(e_{\Omega i})){\underline{\eta }}_{\;\Omega i}^{2p-1}}{{\underline{r}}_{\;\Omega i}(1-{\underline{\eta }}_{\;\Omega i}^{2p})}e_{\Omega i}\frac{{\dot{\underline{r}}}_{\;\Omega i}}{{\underline{r}}_{\;\Omega i}}]. \end{array}$$

The nominal virtual control signals are designed as

(39) $$\omega_{\mathrm{ref},k}=g_{a}^{-1}\left\{\begin{array}{l} -(\kappa_{1,k}+{\bar{\kappa }}_{11,k})e_{\Omega 1}-f_{a1,k}+{\dot{\Omega }}_{\mathrm{ref}1}-{\mathrm{sat}}_{N11,k}({\hat{d}}_{a1,k})-\frac{\eta_{\;\Omega 1}^{2p}}{2\kappa_{2,k}e_{\Omega 1}(1-\eta_{\;\Omega 1}^{2p})}-\frac{2p-1}{2p}e_{\Omega 1}\\ -(\kappa_{1,k}+{\bar{\kappa }}_{12,k})e_{\Omega 2}-f_{a2,k}+{\dot{\Omega }}_{\mathrm{ref}2}-{\mathrm{sat}}_{N12,k}({\hat{d}}_{a2,k})-\frac{\eta_{\;\Omega 2}^{2p}}{2\kappa_{2,k}e_{\Omega 2}(1-\eta_{\;\Omega 2}^{2p})}-\frac{2p-1}{2p}e_{\Omega 2}\\ -(\kappa_{1,k}+{\bar{\kappa }}_{13,k})e_{\Omega 3}-f_{a3,k}+{\dot{\Omega }}_{\mathrm{ref}3}-{\mathrm{sat}}_{N13,k}({\hat{d}}_{a3,k})-\frac{\eta_{\;\Omega 3}^{2p}}{2\kappa_{2,k}e_{\Omega 3}(1-\eta_{\;\Omega 3}^{2p})}-\frac{2p-1}{2p}e_{\Omega 3} \end{array}\right\},$$

where $\kappa_{1,\;k}$ and $\kappa_{2,\;k}$ are both positive constants to be selected. The determinant of $g_{a}$ is −secβ which cannot be zero, because the sideslip β stays in $\left(-\frac{\pi }{2},\;\frac{\pi }{2}\right)$. Considering the definition of $\eta_{\;\Omega i}^{2p}$ as in Equation (36), $\frac{\eta_{\;\Omega i}^{2p}}{e_{\Omega i}}=o(e_{\Omega i})\frac{e_{\Omega i}^{2p-1}}{{\bar{r}}_{\;\Omega i}^{2p-1}}+(1-o(e_{\Omega i}))\frac{e_{\Omega i}^{2p-1}}{{\underline{r}}_{\;\Omega i}^{2p-1}}$, where ${\bar{r}}_{\;\Omega i}^{2p-1}$ and ${\underline{r}}_{\;\Omega i}^{2p-1}$ are both positive. $1-\eta_{\;\Omega i}^{2p}$ is positive under the premise $\left|\eta_{\Omega i}\right|<1$. Then, the nonsingularity of Equation (39) can be guaranteed. The time-varying gain has the following form

(40) $${\bar{\kappa }}_{1i,\;k}(t)=\sqrt{{\left(\frac{{\dot{\bar{r}}}_{\;\Omega i}}{{\bar{r}}_{\;\Omega i}}\right)}^{2}+{\left(\frac{{\dot{\underline{r}}}_{\;\Omega i}}{{\underline{r}}_{\;\Omega i}}\right)}^{2}+\kappa_{3,\;k}},\;i=1,\;2,\;3.$$

where $\kappa_{3,\;k}$ is a positive constant to be designed, and ${\mathrm{sat}}_{N1i,\;k}(\cdot )$ is an odd saturation function defined as

$${\mathrm{sat}}_{N1i,\;k}(x)=\left\{ \begin{array}{ll} x, & 0\leq x\;\leq N1i,\;k,\\ -\frac{1}{2}x^{2}+(N1i,\;k+1)x-\frac{1}{2}m^{2}, & N1i,\;k<x\leq N1i,\;k+1,\\ N1i,\;k+\frac{1}{2}, & x>N1i,\;k+1. \end{array}\right.$$

where i=1, 2, 3 and k∈Ξ.

Introduce the modified dynamic surface technology to derive the derivative of virtual control. The modified first-order filter is designed as

(41) $$\tau_{1,\;k}{\dot{\bar{\omega }}}_{\mathrm{ref}i}+{\bar{\omega }}_{\mathrm{ref}i}=\omega_{\mathrm{ref}i}-\tau_{1,\;k}{\bar{g}}_{ai}\frac{\eta_{\;\Omega i}^{2p}}{e_{\Omega i}(1-\eta_{\;\Omega i}^{2p})}\;,\;i=1,\;2,\;3,$$

where $\tau_{1,\;k}$ is a time constant and ${\bar{g}}_{ai}$ is the sum of the terms on the i-th column of $g_{a}$.

Define the first-order filter error as $z_{1i}={\bar{\omega }}_{\mathrm{ref}i}-\omega_{\mathrm{ref}i}$, and the virtual tracking error as $e_{\omega i}=\omega_{i}-{\bar{\omega }}_{\mathrm{ref}i}$ for i=1, 2, 3. Substituting Equation (39) into Equation (38), one can arrive at

(42) $$\begin{array}{l} {\dot{V}}_{\Omega }=\sum_{i=1}^{3}[\frac{\eta_{\;\Omega i}^{2p}}{e_{\Omega i}(1-\eta_{\;\Omega i}^{2p})}\left(-\kappa_{1}e_{\Omega i}-{\bar{\kappa }}_{1i}e_{\Omega i}-\frac{2p-1}{2p}e_{\Omega i}+{\left(g_{a}e_{\omega }\right)}_{i}+{\left(g_{a}z_{1}\right)}_{i}+{\bar{d}}_{ai.k}-\frac{\eta_{\;\Omega i}^{2p}}{2\kappa_{2}e_{\Omega i}(1-\eta_{\;\Omega i}^{2p})}\right)\\ \;-\frac{o(e_{\Omega i}){\bar{\eta }}_{\;\Omega i}^{2p-1}}{{\bar{r}}_{\;\Omega i}(1-{\bar{\eta }}_{\;\Omega i}^{2p})}e_{\Omega i}\frac{{\dot{\bar{r}}}_{\;\Omega i}}{{\bar{r}}_{\;\Omega i}}-\frac{(1-o(e_{\Omega i})){\underline{\eta }}_{\;\Omega i}^{2p-1}}{{\underline{r}}_{\;\Omega i}(1-{\underline{\eta }}_{\;\Omega i}^{2p})}e_{\Omega i}\frac{{\dot{\underline{r}}}_{\;\Omega i}}{{\underline{r}}_{\;\Omega i}}], \end{array}$$

where ${\bar{d}}_{ai,k}=d_{ai,k}-{\mathrm{sat}}_{N1i}({\hat{d}}_{ai,k})$ for i=1, 2, 3.

Define a compact set $\Pi_{\mathrm{ref}}=\left\{{\left[\Omega_{\mathrm{ref}i},\;{\dot{\Omega }}_{\mathrm{ref}i},\;{\ddot{\Omega }}_{\mathrm{ref}i}\right]}^{T}:\;\Omega_{\mathrm{ref}i}^{2}+{\dot{\Omega }}_{\mathrm{ref}i}^{2}+{\ddot{\Omega }}_{\mathrm{ref}i}^{2}\leq \delta_{\mathrm{ref}}\right\}\subset {\mathbb{R}}^{3}$, where $\delta_{\mathrm{ref}}$ is a positive constant. Define a compact set $\Pi_{\Omega }=\left\{{\left[e_{\Omega 1},\;e_{\Omega 2},\;e_{\Omega 3},\;z_{11},\;z_{12},\;z_{13}\right]}^{T}:\;V_{\Omega }+\frac{1}{2}{\left\|z_{1}\right\|}^{2}\leq \delta_{\Omega }\right\}$, where $\delta_{\Omega }$ is a positive constant.

Obviously, $\omega_{\mathrm{ref}i,k}$ is a continuously differentiable function of $\Omega_{i}$, $\Omega_{\mathrm{ref}i}$, ${\dot{\Omega }}_{\mathrm{ref}i}$, ${\hat{d}}_{ai,k}$, ${\bar{h}}_{\Omega i}$, ${\dot{\bar{h}}}_{\Omega i}$, ${\underline{h}}_{\;\Omega i}$, and ${\dot{\underline{h}}}_{\;\Omega i}$ for i=1, 2, 3. For $t\in [t_{\Omega }+t_{k},\;\infty )$, the ESO designed in Equation (33) becomes the form of Equation (31), where $t_{k}$ is the time when switching to the k-th subsystem. Hence, ${\hat{d}}_{ai,k}$ is continuously differentiable. Under the premise of $\left|\eta_{\Omega i}\right|<1$, we get $-{\underline{r}}_{\;\Omega i}(t)<e_{\Omega i}^{}(t)<{\bar{r}}_{\;\Omega i}(t)$. Noting that $\left|\Omega_{i}\right|=\left|e_{\Omega i}+\Omega_{\mathrm{ref}i}\right|\leq \left|e_{\Omega i}\right|+\left|\Omega_{\mathrm{ref}i}\right|$ and considering Assumptions 1, 3, $\Omega_{i}$ is bounded. Combined with the boundedness of ${\mathrm{sat}}_{N1i,k}({\hat{d}}_{ai,k})$, we obtain that $\omega_{\mathrm{ref}i,k}$ is bounded and moreover assumed to be $\max\left|\omega_{\mathrm{ref}i,k}\right|=D_{\omega i}$, where $D_{\omega i}$ is a positive constant.

The time derivative of $\omega_{\mathrm{ref}i,\;k}$ can be computed as

$${\dot{\omega }}_{\mathrm{ref}i,k}=B(e_{\Omega i},\;z_{1i},\;d_{ai,k}-{\mathrm{sat}}_{N1i}({\hat{d}}_{ai,k}),\;\Omega_{\mathrm{ref}i},\;{\dot{\Omega }}_{\mathrm{ref}i},\;{\ddot{\Omega }}_{\mathrm{ref}i})$$

where B(⋅) is a continuous function. It can be verified that ${\dot{\omega }}_{\mathrm{ref}i,k}$ is bounded on $\Pi_{\mathrm{ref}}\times \Pi_{\Omega }$ and assumed to be $\left|{\dot{\omega }}_{\mathrm{ref}i,k}\right|\leq D_{d\omega i}$ for i=1, 2, 3, where $D_{d\omega i}$ is a positive constant. From Equation (41) we have ${\dot{\bar{\omega }}}_{\mathrm{ref}i}=-z_{1i}/\tau_{1,\;k}-{\bar{g}}_{ai}\eta_{\;\Omega i}^{2p}/[e_{\Omega i}(1-\eta_{\;\Omega i}^{2p})],\;i=1,\;2,\;3,$ then ${\dot{\bar{\omega }}}_{\mathrm{ref}i}$ is bounded on $\Pi_{\mathrm{ref}}\times \Pi_{\Omega }$.

Combined with Assumption 2, we can get

(43) $$\left|{\bar{d}}_{ai,k}\right|\leq \left|d_{ai,k}-{\hat{d}}_{ai,k}\right|+\left|{\hat{d}}_{ai,k}-{\mathrm{sat}}_{N1i,\;k}({\hat{d}}_{ai,k})\right|\leq 2\left|{\tilde{d}}_{ai,k}\right|,\;i=1,\;2,\;3.$$

With the help of Young’s inequality, it can be verified that

(44) $$\frac{\eta_{\;\Omega i}^{2p}{\bar{d}}_{ai,k}^{}}{e_{\Omega i}(1-\eta_{\;\Omega i}^{2p})}\leq \frac{1}{2\kappa_{2,\;k}}{\left(\frac{\eta_{\;\Omega i}^{2p}}{e_{\Omega i}(1-\eta_{\;\Omega i}^{2p})}\right)}^{2}+\frac{\kappa_{2,\;k}}{2}{\bar{d}}_{ai,k}^{2}.$$

Considering the definition of ${\bar{\kappa }}_{1i}(t)$ in Equation (40), it can be noted that

(45) $${\bar{\kappa }}_{1i,\;k}+o(e_{\Omega i})\frac{{\dot{\bar{r}}}_{\;\Omega i}}{{\bar{r}}_{\;\Omega i}}+(1-o(e_{\Omega i}))\frac{{\dot{\underline{r}}}_{\;\Omega i}}{{\underline{r}}_{\;\Omega i}}>0,\;i=1,\;2,\;3.$$

Substituting Equations (43)–(45) into Equation (42) yields

(46) $$\begin{array}{l} {\dot{V}}_{\Omega }\leq \sum_{i=1}^{3}\left[\frac{\eta_{\;\Omega i}^{2p}}{e_{\Omega i}(1-\eta_{\;\Omega i}^{2p})}\left(-\kappa_{1,\;k}e_{\Omega i}-\frac{2p-1}{2p}e_{\Omega i}+{\left(g_{a}e_{\omega }\right)}_{i}+{\left(g_{a}z_{1}\right)}_{i}\right)+2\kappa_{2,\;k}{\tilde{d}}_{ai,k}^{2}\right]\\ \;=\sum_{i=1}^{3}\left[\frac{-\kappa_{1,\;k}\eta_{\;\Omega i}^{2p}}{\left(1-\eta_{\;\Omega i}^{2p}\right)}+2\kappa_{2,\;k}{\tilde{d}}_{ai,k}^{2}-\frac{2p-1}{2p}\beta_{1i}e_{\Omega i}^{2}+\beta_{1i}e_{\Omega i}^{2p-1}\left({\left(g_{a}e_{\omega }\right)}_{i}+{\left(g_{a}z_{1}\right)}_{i}\right)\right]. \end{array}$$

where $\beta_{1i}=\frac{o(e_{\Omega i})}{{\bar{r}}_{\;\Omega i\;}^{2p}-e_{\Omega i}^{2p}}+\frac{1-o(e_{\Omega i})}{{\underline{r}}_{\;\Omega i\;}^{2p}-e_{\Omega i}^{2p}}$.

Utilizing Young’s inequality, we can get

(47) $$\beta_{1i}e_{\Omega i}^{2p-1}{\left(g_{a}e_{\omega }\right)}_{i}\leq \beta_{1i}\left[\frac{2p-1}{2p}e_{\Omega i}^{2p}+\frac{1}{2p}{\left(g_{a}e_{\omega }\right)}_{i}^{2p}\right],\;i=1,\;2,\;3.$$

Then, Equation (45) can be rewritten as

(48) $${\dot{V}}_{\Omega }=\sum_{i=1}^{3}\left[\frac{-\kappa_{1,\;k}\eta_{\;\Omega i}^{2p}}{\left(1-\eta_{\;\Omega i}^{2p}\right)}+2\kappa_{2,\;k}{\tilde{d}}_{ai,k}^{2}+\beta_{1i}e_{\Omega i}^{2p-1}{\left(g_{a}z_{1}\right)}_{i}+\frac{\beta_{1i}}{2p}{\left(g_{a}e_{\omega }\right)}_{i}^{2p}\right].$$

### 4.2. Control Law Design for Attitude Angular Rate System

Considering the second equation in (1), choose $d_{v,k}^{}$ as the extended state, and introduce the ESO as in Equation (3) to estimate the total disturbances.

(49) $$\left\{ \begin{array}{l} \dot{\hat{\omega }}=f_{v,k}^{}+g_{v,k}^{}M_{v}^{}+{\hat{d}}_{v,k}^{}+\lambda_{1\omega ,\;k}\left[\frac{\omega -\hat{\omega }}{\varepsilon_{1\omega ,\;k}}+\lambda_{3\omega ,\;k}(\frac{\varepsilon_{1\omega ,\;k}}{\varepsilon_{2\omega ,\;k}}-1)\cdot h(\frac{\omega -\hat{\omega }}{\varepsilon_{1\omega ,\;k}\lambda_{3\omega ,\;k}})\right],\\ {\dot{\hat{d}}}_{v,k}^{}=\lambda_{2\omega ,\;k}\left[\frac{\omega -\hat{\omega }}{\varepsilon_{1\omega ,\;k}^{2}}+\lambda_{3\omega ,\;k}(\frac{\varepsilon_{1\omega ,\;k}}{\varepsilon_{2\omega ,\;k}^{2}}-\frac{1}{\varepsilon_{1\omega ,\;k}})\cdot h(\frac{\omega -\hat{\omega }}{\varepsilon_{1\omega ,\;k}\lambda_{3\omega ,\;k}})\right], \end{array}\right.$$

where $\lambda_{1\omega ,k}$, $\lambda_{2\omega ,k}$, $\lambda_{3\omega ,\;k}$, $\varepsilon_{1\omega ,\;k}$, and $\varepsilon_{2\omega ,\;k}$ are positive constants to be designed, such that $\varepsilon_{1\omega ,\;k}<\varepsilon_{2\omega ,\;k}\ll 1$.

On the basis of Theorem 1 and Assumption 2, it can be concluded that there exist $\lambda_{1\omega ,k}$, $\lambda_{2\omega ,k}$, $\lambda_{3\omega ,\;k}$, $\varepsilon_{1\omega ,\;k}$, and $\varepsilon_{2\omega ,\;k}$ such that the estimation errors $\left\|\omega -\hat{\omega }\right\|$ and $\left\|d_{v,k}^{}-{\hat{d}}_{v,k}^{}\right\|$ will converge to a small region of zero for $t\in [t_{\omega ,\;k},\;+\infty )$, where $t_{\omega }$ is a positive constant determined by $\varepsilon_{1\omega ,\;k}$ and $\varepsilon_{2\omega ,\;k}$. Specifically, we suppose $\left\|{\tilde{d}}_{v,k}^{}\right\|=\left\|d_{v,k}^{}-{\hat{d}}_{v,k}^{}\right\|\leq D_{v,\;k}$ for $t\in [t_{\omega ,\;k},\;+\infty )$.

Define the attitude angular rate tracking error as $e_{\omega i}=\omega_{i}-{\bar{\omega }}_{\mathrm{ref}i}$ for i=1, 2, 3. The dynamics of $e_{\omega i}$ is

(50) $${\dot{e}}_{\omega i}=f_{vi,\;k}^{}+{\left(g_{v,\;k}^{}M_{v}^{}\right)}_{i}+d_{vi,\;k}^{}-{\dot{\bar{\omega }}}_{\mathrm{ref}i}$$

Choose the time-varying asymmetric BLF candidate as

(51) $$V_{\omega }=\sum_{i=1}^{3}\frac{o(e_{\omega i})}{2p}\log\frac{{\bar{r}}_{\;\omega i}^{2p}}{{\bar{r}}_{\;\omega i}^{2p}-e_{\omega i}^{2p}}+\frac{1-o(e_{\omega i})}{2p}\log\frac{{\underline{r}}_{\;\omega i}^{2p}}{{\underline{r}}_{\;\omega i}^{2p}-e_{\omega i}^{2p}},$$

where ${\bar{r}}_{\;\omega i}^{}$ and ${\underline{r}}_{\;\omega i}^{}$ will be specified later on.

Introduce change of coordinate as

(52) $$\left\{ \begin{array}{l} {\bar{\eta }}_{\omega i}^{}=\frac{e_{\omega i}}{{\bar{r}}_{\;\omega i}^{}},\;{\underline{\eta }}_{\Omega i}^{}=\frac{e_{\omega i}}{{\underline{r}}_{\;\omega i}^{}},\\ \eta_{\omega i}=o(e_{\omega i}){\bar{\eta }}_{\omega i}^{}+(1-o(e_{\omega i})){\underline{\eta }}_{\omega i}^{}. \end{array}\right.$$

Then, Equation (51) can be rewritten as

(53) $$V_{\omega }=\sum_{i=1}^{3}\frac{1}{2p}\log\frac{1}{1-\eta_{\omega i}^{2p}}.$$

It is clear that under the premise of $\left|\eta_{\omega i}\right|<1$, $V_{\omega }$ is a rational Lyapunov candidate which is positive definite and continuously differentiable.

Consider Equation (50) and the derivative of $V_{\omega }$ can be written as

(54) $$\begin{array}{l} {\dot{V}}_{\omega }=\sum_{i=1}^{3}\left[\frac{o(e_{\omega i}){\bar{\eta }}_{\;\omega i}^{2p-1}}{{\bar{r}}_{\;\omega i}(1-{\bar{\eta }}_{\;\omega i}^{2p})}\left({\dot{e}}_{\omega i}-e_{\omega i}\frac{{\dot{\bar{r}}}_{\;\omega i}}{{\bar{r}}_{\;\omega i}}\right)+\frac{(1-o(e_{\omega i})){\underline{\eta }}_{\;\omega i}^{2p-1}}{{\underline{r}}_{\;\omega i}(1-{\underline{\eta }}_{\;\omega i}^{2p})}\left({\dot{e}}_{\omega i}-e_{\omega i}\frac{{\dot{\underline{r}}}_{\;\omega i}}{{\underline{r}}_{\;\omega i}}\right)\right]\\ \;=\sum_{i=1}^{3}[\frac{\eta_{\;\omega i}^{2p}}{e_{\omega i}(1-\eta_{\;\omega i}^{2p})}\left(f_{vi,\;k}^{}+{\left(g_{v,\;k}^{}M_{v}^{}\right)}_{i}+d_{vi,\;k}^{}-{\dot{\bar{\omega }}}_{\mathrm{ref}i}\right)\\ \;-\frac{o(e_{\omega i}){\bar{\eta }}_{\;\omega i}^{2p-1}}{{\bar{r}}_{\;\omega i}(1-{\bar{\eta }}_{\;\omega i}^{2p})}e_{\omega i}\frac{{\dot{\bar{r}}}_{\;\omega i}}{{\bar{r}}_{\;\omega i}}-\frac{(1-o(e_{\omega i})){\underline{\eta }}_{\;\omega i}^{2p-1}}{{\underline{r}}_{\;\omega i}(1-{\underline{\eta }}_{\;\omega i}^{2p})}e_{\omega i}\frac{{\dot{\underline{r}}}_{\;\omega i}}{{\underline{r}}_{\;\omega i}}]. \end{array}$$

The actual controller can be designed as follows

(55) $$\begin{array}{l} \;M_{v,\;k}^{}\\ =g_{v,k}^{-1}\left\{\begin{array}{l} -(\kappa_{4,\;k}+{\bar{\kappa }}_{21,\;k})e_{\omega 1}-f_{v1,\;k}+{\dot{\bar{\omega }}}_{\mathrm{ref}1}-{\mathrm{sat}}_{N21,\;k}({\hat{d}}_{v1,k})-\frac{\eta_{\;\omega 1}^{2p}}{2\kappa_{5,\;k}e_{\omega 1}(1-\eta_{\;\omega 1}^{2p})}-\frac{\beta_{11}}{2pe_{\omega 1}^{2p-1}\beta_{21}}{\left(g_{a}e_{\omega }\right)}_{1}^{2p}\\ -(\kappa_{4,\;k}+{\bar{\kappa }}_{22,\;k})e_{\omega 2}-f_{a2,\;k}+{\dot{\bar{\omega }}}_{\mathrm{ref}2}-{\mathrm{sat}}_{N22,\;k}({\hat{d}}_{v2,k})-\frac{\eta_{\;\omega 2}^{2p}}{2\kappa_{5,\;k}e_{\omega 2}(1-\eta_{\;\omega 2}^{2p})}-\frac{\beta_{12}}{2pe_{\omega 2}^{2p-1}\beta_{22}}{\left(g_{a}e_{\omega }\right)}_{2}^{2p}\\ -(\kappa_{4,\;k}+{\bar{\kappa }}_{23,\;k})e_{\omega 3}-f_{a3,\;k}+{\dot{\bar{\omega }}}_{\mathrm{ref}3}-{\mathrm{sat}}_{N23,\;k}({\hat{d}}_{v3,k})-\frac{\eta_{\;\omega 3}^{2p}}{2\kappa_{5,\;k}e_{\omega 3}(1-\eta_{\;\omega 3}^{2p})}-\frac{\beta_{13}}{2pe_{\omega 3}^{2p-1}\beta_{23}}{\left(g_{a}e_{\omega }\right)}_{3}^{2p} \end{array}\right\}, \end{array}$$

where $\kappa_{4,\;k}$ and $\kappa_{5,\;k}$ are positive constants, $\beta_{2i}=\frac{o(e_{\omega i})}{{\bar{r}}_{\;\omega i\;}^{2p}-e_{\omega i}^{2p}}+\frac{1-o(e_{\omega i})}{{\underline{r}}_{\;\omega i\;}^{2p}-e_{\omega i}^{2p}}$ for i=1, 2, 3, and ${\bar{\kappa }}_{2i,\;k}$ is the time-varying gain, shown as

(56) $${\bar{\kappa }}_{2i,\;k}(t)=\sqrt{{\left(\frac{{\dot{\bar{r}}}_{\;\omega i}}{{\bar{r}}_{\;\omega i}}\right)}^{2}+{\left(\frac{{\dot{\underline{r}}}_{\;\omega i}}{{\underline{r}}_{\;\omega i}}\right)}^{2}+\kappa_{6,\;k}},\;i=1,\;2,\;3,$$

where $\kappa_{6,\;k}$ is a positive constant.

Noting ${\bar{\kappa }}_{2i,\;k}+o(e_{\omega i})\frac{{\dot{\bar{r}}}_{\;\omega i}}{{\bar{r}}_{\;\omega i}}+(1-o(e_{\omega i}))\frac{{\dot{\underline{r}}}_{\;\omega i}}{{\underline{r}}_{\;\omega i}}>0$ for i=1, 2, 3 and taking Equation (55) into Equation (54) leads to

(57) $$\begin{array}{l} {\dot{V}}_{\omega }=\sum_{i=1}^{3}[\frac{-(\kappa_{4,\;k}+{\bar{\kappa }}_{21,\;k})\eta_{\;\omega i}^{2p}}{\left(1-\eta_{\;\omega i}^{2p}\right)}+\frac{\eta_{\;\omega i}^{2p}}{e_{\omega i}(1-\eta_{\;\omega i}^{2p})}\left({\bar{d}}_{vi,k}-\frac{\eta_{\;\omega i}^{2p}}{2\kappa_{5,\;k}e_{\omega i}(1-\eta_{\;\omega i}^{2p})}-\frac{\beta_{1i}}{2pe_{\omega i}^{2p-1}\beta_{2i}}{\left(g_{a}e_{\omega }\right)}_{i}^{2p}\right)\\ \;-\frac{o(e_{\omega i}){\bar{\eta }}_{\;\omega i}^{2p-1}}{{\bar{r}}_{\;\omega i}(1-{\bar{\eta }}_{\;\omega i}^{2p})}e_{\omega i}\frac{{\dot{\bar{r}}}_{\;\omega i}}{{\bar{r}}_{\;\omega i}}-\frac{(1-o(e_{\omega i})){\underline{\eta }}_{\;\omega i}^{2p-1}}{{\underline{r}}_{\;\omega i}(1-{\underline{\eta }}_{\;\omega i}^{2p})}e_{\Omega i}\frac{{\dot{\underline{r}}}_{\;\omega i}}{{\underline{r}}_{\;\omega i}}]\\ \;\leq \sum_{i=1}^{3}\left[\frac{-\kappa_{4,\;k}\eta_{\;\omega i}^{2p}}{\left(1-\eta_{\;\omega i}^{2p}\right)}+\frac{\eta_{\;\omega i}^{2p}}{e_{\omega i}(1-\eta_{\;\omega i}^{2p})}\left({\bar{d}}_{vi,k}-\frac{\eta_{\;\omega i}^{2p}}{2\kappa_{5,\;k}e_{\omega i}(1-\eta_{\;\omega i}^{2p})}\right)-\frac{\beta_{1i}}{2p}{\left(g_{a}e_{\omega }\right)}_{i}^{2p}\right], \end{array}$$

where ${\bar{d}}_{vi,k}=d_{vi,k}-{\mathrm{sat}}_{N2i,\;k}({\hat{d}}_{vi,k})$ for i=1, 2, 3.

Considering Assumption 2, we have

(58) $${\bar{d}}_{vi,k}\leq \left|d_{vi,k}-{\hat{d}}_{vi,k}\right|+\left|{\hat{d}}_{vi,k}-{\mathrm{sat}}_{N1i,\;k}({\hat{d}}_{vi,k})\right|\leq 2\left|{\tilde{d}}_{vi,k}\right|,\;i=1,\;2,\;3.$$

Combined with Young’s inequality, the following inequality holds:

(59) $$\frac{\eta_{\;\omega i}^{2p}{\bar{d}}_{vi,k}^{}}{e_{\omega i}(1-\eta_{\;wi}^{2p})}\leq \frac{1}{2\kappa_{5,\;k}}{\left(\frac{\eta_{\;wi}^{2p}}{e_{wi}(1-\eta_{\;wi}^{2p})}\right)}^{2}+\frac{\kappa_{5,\;k}}{2}{\bar{d}}_{vi,k}^{2}.$$

Substituting Equations (58) and (59) into Equation (57) yields

(60) $${\dot{V}}_{\omega }\leq \sum_{i=1}^{3}\left[\frac{-\kappa_{4,\;k}\eta_{\;\omega i}^{2p}}{\left(1-\eta_{\;\omega i}^{2p}\right)}+2\kappa_{5,\;k}{\tilde{d}}_{vi,k}^{2}-\frac{\beta_{1i}}{2p}{\left(g_{a}e_{\omega }\right)}_{i}^{2p}\right].$$

## 5. Stability and Performance Analysis

In this section, the stability of the tracking error is discussed. Define a compact set $\Pi_{e}=\left\{\left|\eta_{\;\Omega i}^{}\right|<1,\;\left|\eta_{\;\omega i}^{}\right|<1,\;i=1,\;2,\;3\right\}$, and we need the following lemma to assist in completing the proof.

**Lemma** **3** **[20]**. *The condition*$\left|\eta_{\;\Omega i}^{}\right|<1$*holds true if and only if*$-{\underline{r}}_{\;\Omega i}(t)<e_{\Omega i}^{}(t)<{\bar{r}}_{\;\Omega i}(t)$*,*$\left|\eta_{\;\omega i}^{}\right|<1$*holds true if and only if*$-{\underline{r}}_{\;\omega i}(t)<e_{\omega i}^{}(t)<{\bar{r}}_{\;\omega i}(t)$*, for*i=1, 2, 3*.*

**Theorem** **2.** *Consider the VSNSV attitude motion formed of the closed-loop nonlinear switched system (1), the extended state observers Equation (33), Equation (49), the virtual control input Equation (39), the control signal Equation (55), and the modified first-order filters (41) and that Assumptions 1–3 are satisfied. For bounded initial conditions, satisfy that* ${\underline{h}}_{\Omega i}(0)<\Omega_{i}(0)<{\bar{h}}_{\Omega i}(0)$ *,* ${\underline{h}}_{\omega i}(0)<\omega_{i}<{\bar{h}}_{\omega i}(0)$ *,* $-{\underline{r}}_{\;\omega i}^{}(0)<e_{\omega i}^{}(0)<{\bar{r}}_{\;\omega i}^{}(0)$ *for* i=1, 2, 3 *, and the proposed control scheme guarantees the following characteristics:*

(1) The tracking errors $e_{\Omega i}(t)$ and $e_{\omega i}(t)$ are bounded by $-{\underline{E}}_{\;\Omega i}(t)\leq e_{\Omega i}(t)\leq {\bar{E}}_{\;\Omega i}(t)$ and $-{\underline{E}}_{\;\omega i}(t)\leq e_{\omega i}(t)\leq {\bar{E}}_{\;\omega i}(t)$ for i=1, 2, 3, where ${\underline{E}}_{\;\Omega i}(t)$, ${\bar{E}}_{\;\Omega i}(t)$, ${\underline{E}}_{\;\omega i}(t)$, and ${\bar{E}}_{\;\omega i}(t)$ will be defined later on.

(2) The asymmetric time-varying state constraints are not violated, such as ${\underline{h}}_{\Omega i}(t)\leq \Omega_{i}\leq {\bar{h}}_{\Omega i}(t)$, ${\underline{h}}_{\omega i}(t)\leq \omega_{i}\leq {\bar{h}}_{\omega i}(t)$, for i=1, 2, 3.

(3) All signals in the closed loop are bounded.

(4) The system output tracking errors converge to a desired neighborhood of zero.

**Proof.**  For the *k*-th subsystem of VSNSV, consider the Lyapunov candidate as follows

(61) $$\begin{array}{l} V=V_{\Omega }+V_{\omega }+\frac{1}{2}{\left\|z_{1}\right\|}^{2}\\ \;=\frac{1}{2p}\sum_{i=1}^{3}\left(\log\frac{1}{1-\eta_{\;\Omega i}^{2p}}+\log\frac{1}{1-\eta_{\omega i}^{2p}}\right)+\frac{1}{2}{\left\|z_{1}\right\|}^{2}. \end{array}$$

 □

Combined with Equations (41), (48) and (60), and compute the time derivate of V as

(62) $$\begin{array}{l} \dot{V}={\dot{V}}_{\Omega }+{\dot{V}}_{\omega }+\sum_{i=1}^{3}z_{1i}{\dot{z}}_{1i}\\ \;\leq \sum_{i=1}^{3}\left[\frac{-\kappa_{1,\;k}\eta_{\;\Omega i}^{2p}}{\left(1-\eta_{\;\Omega i}^{2p}\right)}+2\kappa_{2,\;k}{\tilde{d}}_{ai,k}^{2}+\beta_{1i}e_{\Omega i}^{2p-1}{\left(g_{a}z_{1}\right)}_{i}+\frac{\beta_{1i}}{2p}{\left(g_{a}e_{\omega }\right)}_{i}^{2p}\right]\\ \;+\sum_{i=1}^{3}\left[\frac{-\kappa_{4,\;k}\eta_{\;\omega i}^{2p}}{\left(1-\eta_{\;\omega i}^{2p}\right)}+2\kappa_{5,\;k}{\tilde{d}}_{vi,k}^{2}-\frac{\beta_{1i}}{2p}{\left(g_{a}e_{\omega }\right)}_{i}^{2p}\right]\;\\ \;+\sum_{i=1}^{3}z_{1i}\left(\frac{\omega_{\mathrm{ref}i}-{\bar{\omega }}_{\mathrm{ref}i}}{\tau_{1,\;k}}-{\bar{g}}_{ai}\frac{\eta_{\;\Omega i}^{2p}}{e_{\Omega i}(1-\eta_{\;\Omega i}^{2p})}-{\dot{\omega }}_{\mathrm{ref}i}\right) \end{array}$$

$$\leq \sum_{i=1}^{3}\left[\frac{-\kappa_{1,\;k}\eta_{\;\Omega i}^{2p}}{\left(1-\eta_{\;\Omega i}^{2p}\right)}+\frac{-\kappa_{4,\;k}\eta_{\;\omega i}^{2p}}{\left(1-\eta_{\;\omega i}^{2p}\right)}+2\kappa_{2,\;k}{\tilde{d}}_{ai,k}^{2}+2\kappa_{5,\;k}{\tilde{d}}_{vi,k}^{2}-\frac{z_{1i}^{2}}{\tau_{1,\;k}}+D_{d\omega i}\right].$$

Noting that $\left\|{\tilde{d}}_{a,k}^{}\right\|\leq D_{a,\;k}$ and $\left\|{\tilde{d}}_{v,k}^{}\right\|\leq D_{v,\;k}$ for $t\in [t_{k}+t_{e,\;k},\;\infty )$, where $t_{k}$ is the time when switching to the k-th subsystem, $t_{e,\;k}=\max\{t_{\Omega ,\;k},\;t_{\omega ,\;k}\}$.

Then Equation (62) can be simplified as

(63) $$\dot{V}\;\leq \sum_{i=1}^{3}\left[\frac{-\kappa_{1,\;k}\eta_{\;\Omega i}^{2p}}{\left(1-\eta_{\;\Omega i}^{2p}\right)}+\frac{-\kappa_{4,\;k}\eta_{\;\omega i}^{2p}}{\left(1-\eta_{\;\omega i}^{2p}\right)}-\frac{z_{1i}^{2}}{\tau_{1,\;k}}+D_{d\omega i}\right]+2\kappa_{2,\;k}D_{a,\;k}^{2}+2\kappa_{5,\;k}D_{v,\;k}^{2}.$$

Utilizing Lemma 1, we can arrive at

(64) $$\begin{array}{l} \dot{V}\;\leq \sum_{i=1}^{3}\left[-\kappa_{1,\;k}\log\frac{1}{\left(1-\eta_{\;\Omega i}^{2p}\right)}-\kappa_{4,\;k}\log\frac{1}{\left(1-\eta_{\;\omega i}^{2p}\right)}-\frac{z_{1i}^{2}}{\tau_{1,\;k}}+D_{d\omega i}\right]+2\kappa_{2,\;k}D_{a,\;k}^{2}+2\kappa_{5,\;k}D_{v,\;k}^{2}\\ \;\leq -\mu V+C, \end{array}$$

in the set $\Pi_{e}$, where $\mu =\min\left\{2p\kappa_{1,\;k},\;2p\kappa_{4,\;k},\;\frac{2}{\tau_{1,\;k}}\right\}$, $C=2\kappa_{2,\;k}D_{a,\;k}^{2}+2\kappa_{5,\;k}D_{v,\;k}^{2}+\sum_{i=1}^{3}D_{d\omega i}$.

Considering the definition of ${\bar{r}}_{\;\Omega i}^{}$, ${\underline{r}}_{\;\Omega i}^{}$, analyze the initial conditions and we can get $-{\underline{r}}_{\;\Omega i}(0)<e_{\Omega i}^{}(0)<{\bar{r}}_{\;\Omega i}(0)$. Combined with $-{\underline{r}}_{\;\omega i}(0)<e_{\omega i}^{}(0)<{\bar{r}}_{\;\omega i}(0)$, it can be verified that $\left|\eta_{\;\Omega i}^{}(0)\right|<1$ and $\left|\eta_{\;\omega i}^{}(0)\right|<1$ for i=1, 2, 3, as follows from Lemma 3. With the help of Lemma 2, we have $\left|\eta_{\;\Omega i}^{}(t)\right|<1$ and $\left|\eta_{\;\omega i}^{}(t)\right|<1$, ∀t>0 for i=1, 2, 3. Utilizing Lemma 3 again, we can get $-{\underline{r}}_{\;\Omega i}(t)<e_{\Omega i}^{}(t)<{\bar{r}}_{\;\Omega i}(t)$ and $-{\underline{r}}_{\;\omega i}(t)<e_{\omega i}^{}(t)<{\bar{r}}_{\;\omega i}(t)$ ∀t>0 for i=1, 2, 3. Therefore, the condition ${\underline{h}}_{\Omega i}<\Omega_{i}<{\bar{h}}_{\Omega i}$ for i=1, 2, 3 can be guaranteed.

By solving the differential equation in (64), we have

(65) $$V(t)\leq V(0)e^{-\mu t}+\frac{C}{\mu }(1-e^{-\mu t})\leq \bar{V}(0)+\frac{C}{\mu }.$$

where $\bar{V}(0)=\frac{1}{2p}\sum_{i=1}^{3}\left(\log\frac{1}{1-\eta_{\;\Omega i}^{2p}(0)}+\log\frac{1}{1-\eta_{\omega i}^{2p}(0)}\right)+\frac{1}{2}{\left\|z_{1}(0)\right\|}^{2}.$

From the definition of V, we have $(1/2p)\log(1/1-\eta_{\;\Omega i}^{2p})\leq \bar{V}(0)+C/\mu$ and $(1/2p)\log(1/1-\eta_{\;\omega i}^{2p})\leq \bar{V}(0)+C/\mu$. Furthermore, we can get $\eta_{\;\Omega i}^{}\leq {\left(1-e^{-2p(\bar{V}(0)+C/\mu )}\right)}^{1/2p}$ and $\eta_{\;\omega i}^{}\leq {\left(1-e^{-2p(\bar{V}(0)+C/\mu )}\right)}^{1/2p}$. Considering Equation (36) and Equation (52), it can be concluded that $-{\underline{E}}_{\;\Omega i}(t)\leq e_{\Omega i}(t)\leq {\bar{E}}_{\;\Omega i}(t)$ and $-{\underline{E}}_{\;\omega i}(t)\leq e_{\omega i}(t)\leq {\bar{E}}_{\;\omega i}(t)$, where ${\underline{E}}_{\;\Omega i}(t)=$ ${\underline{r}}_{\;\Omega i}{\left(1-e^{-2p(\bar{V}(0)+C/\mu )}\right)}^{1/2p}$, ${\bar{E}}_{\;\Omega i}(t)={\bar{r}}_{\;\Omega i}{\left(1-e^{-2p(\bar{V}(0)+C/\mu )}\right)}^{1/2p}$, ${\underline{E}}_{\;\omega i}(t)={\underline{r}}_{\;\omega i}{\left(1-e^{-2p(\bar{V}(0)+C/\mu )}\right)}^{1/2p}$, and ${\bar{E}}_{\;\omega i}(t)=$ ${\bar{r}}_{\;\omega i}{\left(1-e^{-2p(\bar{V}(0)+C/\mu )}\right)}^{1/2p}$. Moreover, we can prove $\left|z_{1i}\right|\leq Z_{1i}$, where $Z_{1i}=\sqrt{2(\bar{V}(0)+C/\mu )}$ for i=1, 2, 3.

Noting that in the previous analysis in Section 4.1, $\omega_{\mathrm{ref}i,k}$ is bounded with $\left|\omega_{\mathrm{ref}i,k}\right|\leq D_{\omega i}$, $\kappa_{1,k}$, $\kappa_{2,\;k}$, $\kappa_{3,k}$, $\kappa_{4,\;k}$, $\kappa_{5,\;k}$, p and $\tau_{1,\;k}$ should be designed to guarantee ${\underline{h}}_{\omega i}(t)<Z_{1i}+D_{\omega i}<{\bar{h}}_{\omega i}(t)$ for i=1, 2, 3. Noting that $\omega_{i}=e_{\omega i}+z_{1i}+\omega_{\mathrm{ref}i}$ for i=1, 2, 3, ${\underline{h}}_{\omega i}(t)<\omega_{i}<{\bar{h}}_{\omega i}(t)$ is guaranteed as long as ${\bar{r}}_{\;\omega i}^{}={\bar{h}}_{\omega i}(t)-Z_{1i}-D_{\omega i}$, ${\underline{r}}_{\;\omega i}^{}=Z_{1i}+D_{\omega i}-{\underline{h}}_{\omega i}(t)$. Furthermore, considering the control law in Equation (55), the boundedness of $M_{vi,\;k}^{}$ can be guaranteed.

From Equation (65), we further arrive at

(66) $$\eta_{\;\Omega i}^{}\leq {\left(1-e^{-(2p\bar{V}(0)-C/\mu )e^{-\mu t}-C/\mu }\right)}^{\frac{1}{2p}}.$$

Along with t→∞, $\eta_{\;\Omega i}^{}\leq {\left(1-e^{-C/\mu }\right)}^{\frac{1}{2p}}.$ Then the attitude tracking error $e_{\Omega i}$ can be arbitrarily small with appropriate C and μ for i=1, 2, 3. The proof is completed.

**Remark** **6.** *Considering the size of tracking residual and the convergence rate as shown in Equation (66),*μ*should be set to be large enough,*C*should be set as small, and*p*should be a small positive integer. Furthermore, combined with the definition of*μ*, we need to choose a large*$\kappa_{1,\;k}$*and*$\kappa_{4,\;k}$*and a small*$\tau_{1,\;k}$*. Combined with the definition of*C*, a large*$\kappa_{2,\;k}$*and*$\kappa_{5,\;k}$*can increase the convergence rate. Considering Equations(40) and (56),*$\kappa_{3,\;k}$*and*$\kappa_{6,\;k}$ should be positive and large.

**Remark** **7.** *The problem of the ‘explosion of complexity’ is tackled via the dynamic surface control scheme, which uses a modified first-order filter to synthetic input at two steps of controller design. In the generic dynamic surface control scheme, the coupling terms such as*$\beta_{1i}e_{\Omega i}^{2p-1}{\left(g_{a}z_{1}\right)}_{i}$*in Equation (48) and Equation (62) are decoupled with the help of Young’s inequality, which needs the hypothesis that there exists the upper bound of*$\left\|g_{a}\right\|$*[45,46]. The hypothesis seriously increases the conservativeness of controller design. It should be pointed out that in this paper we present a modified dynamic surface, as in Equation (41). The last term in the first-order filter*$\tau_{1,\;k}{\bar{g}}_{ai}[\eta_{\;\Omega i}^{2p}/e_{\Omega i}(1-\eta_{\;\Omega i}^{2p})]$*eliminates the coupling term*$\beta_{1i}e_{\Omega i}^{2p-1}{\left(g_{a}z_{1}\right)}_{i}$*in order to avoid the priori knowledge of*$g_{a}$.

## 6. Numerical Simulation

To confirm the superiority and effectiveness of the proposed controller, a numerical simulation was conducted compared with the approach in [10]. The aerodynamic coefficients of VSNSVs are from [47]. The wing sweep angle Λ changed between $60^{\circ }$ and $75^{\circ }$, and the flight characteristics when $60^{\circ }\leq \Lambda <67^{\circ }$ and $67^{\circ }\leq \Lambda \leq 75^{\circ }$ are described by subsystems $\sigma_{1}$ and $\sigma_{2}$, respectively. The VSNSV was assumed to carry out a flight at the speed of 1250 m/s and at an altitude of 30 km. The initial condition was set as $\alpha =1^{\circ }$, $\beta =-1^{\circ }$, $\mu =1^{\circ }$, $\Lambda =60^{\circ }$, and $p=q=r=0^{\circ }$. During the simulation, Λ varied from $60^{\circ }$ to $75^{\circ }$ and the subsystem $\sigma_{2}$ was activated at t=8 s. Next, Λ varied from $75^{\circ }$ to $60^{\circ }$, and the subsystem switched to $\sigma_{1}$ at t=15 s. To demonstrate the validity of the designed extended state observer, 25% uncertainties of the aerodynamic coefficients were considered. The external disturbances imposed on the attitude angle loop and angular rate loop were set as ${\left[\sin(2t),\;1.5\cos(3t),\;-\cos(t)\right]}^{T}\;/\;(\deg/s)$ and $[4\times 10^{5}\cos(3t),\;5\times 10^{5}\cos(4t),$ $3.5\times 10^{5}\sin(2t){]}^{T}\;N\cdot m$ for the subsystem $\sigma_{1}$, ${\left[2\sin(t),\;\cos(3t),\;-\cos(2t)\right]}^{T}\;/\;(\deg/s)$ and $[3\times 10^{5}\cos(4t),\;2\times 10^{5}\cos(2t),$ $5\times 10^{5}\sin(2t){]}^{T}\;N\cdot m$ for the subsystem $\sigma_{2}$.

The desired outputs were $\alpha_{\mathrm{ref}}=-0.2t^{2}+2t$ for t∈[0, 5), $\alpha_{\mathrm{ref}}=5$ for t∈[5, ∞), $\beta_{\mathrm{ref}}=0$, $\mu_{\mathrm{ref}}=0$. Obviously, the command signal was continuously differentiable. The state constraints were $-0.2t^{2}+2t-0.1\cos(t)-0.5<\alpha <-0.2t^{2}+2t+0.4\cos(t)+0.7$ for t∈[0, 5), 4.5−0.1cos(t)< α<5.7+0.3cos(t) for t∈[5, ∞), $e^{-0.2t}\times [-1\cos(t)-1.5]<\beta <0.05\cos(t)+0.15$, $e^{-0.2t}\times [-1\cos(t)-1.5]<\mu <0.05\cos(t)+0.15$, $e^{-0.2t}\times [0.5\sin(t)+1]<p<e^{-0.2t}\times [-\cos(t)-3]$, −0.1cos(t)−0.5<q<0.3cos(t)+0.8, and −0.1cos(t)−0.5<r<0.3cos(t)+0.8.

Based on the extended state observer designed in Section 3 and the parameter selection principle in Remark 5, the corresponding parameters were chosen as $\lambda_{1\Omega }=2$, $\lambda_{2\Omega }=1$, $\lambda_{3\Omega }=8$, $\varepsilon_{1\Omega }=0.02$, $\varepsilon_{2\Omega }=0.1$ for the attitude angle system ESO, $\lambda_{1\omega }=4$, $\lambda_{2\omega }=4$, $\lambda_{3\omega }=15$, $\varepsilon_{1\omega }=0.02$, $\varepsilon_{2\omega }=0.1$ for the angular rate system ESO. The ESO parameters for subsystems $\sigma_{1}$ and $\sigma_{2}$ were the same. According to the control scheme proposed in Remark 6, $\kappa_{1,\;\sigma_{1}}=6$, $\kappa_{2,\;\sigma_{1}}=4$, $\kappa_{3,\;\sigma_{1}}=5$, $\kappa_{4,\;\sigma_{1}}=8$, $\kappa_{5,\;\sigma_{1}}=5$, $\kappa_{6,\;\sigma_{1}}=5$ and $\kappa_{1,\;\sigma_{2}}=8$, $\kappa_{2,\;\sigma_{2}}=7$, $\kappa_{3,\;\sigma_{2}}=5$, $\kappa_{4,\;\sigma_{2}}=10$, $\kappa_{5,\;\sigma_{2}}=8$, $\kappa_{6,\;\sigma_{2}}=5$. The first-order filter was designed with the time constant $\tau_{1}=0.1$ for the two subsystems.

**Figure 2:**
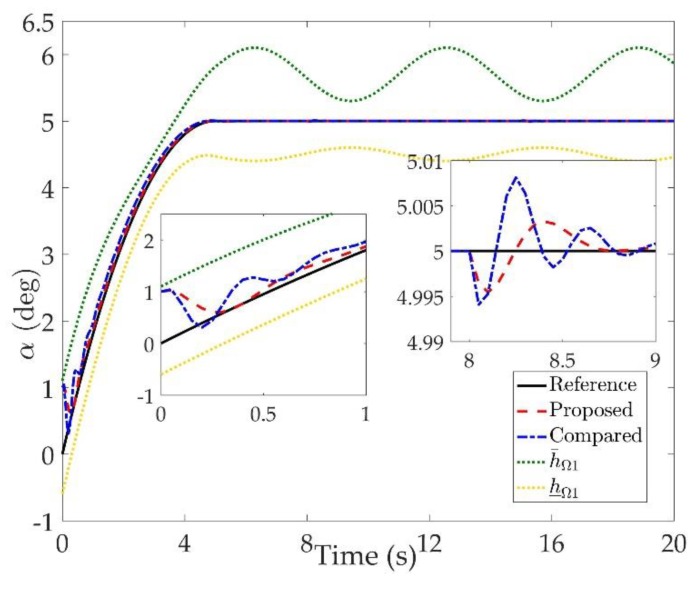
Comparison curves of the angle of attack α tracking performance.

**Figure 3:**
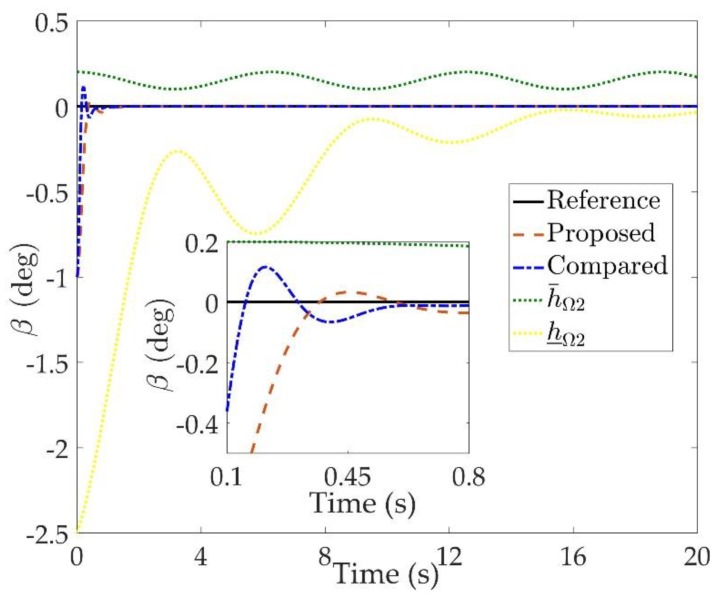
Comparison curves of the sideslip β tracking performance.

**Figure 4:**
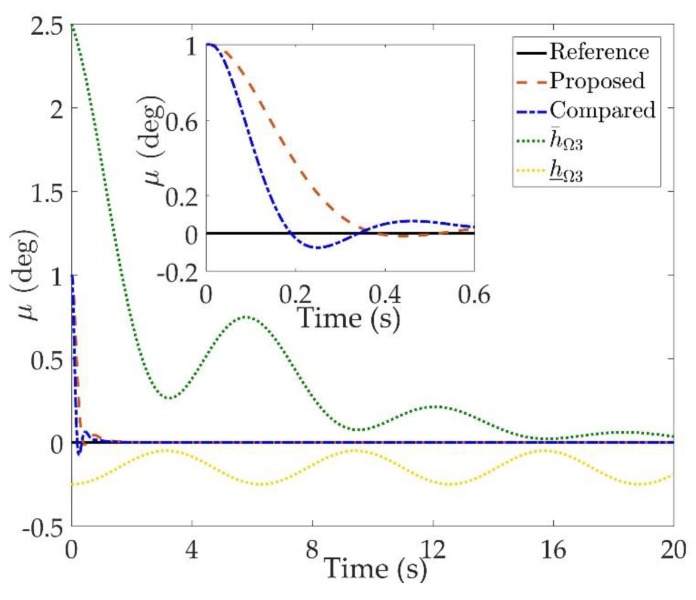
Comparison curves of the bank angle μ tracking performance.

**Figure 5:**
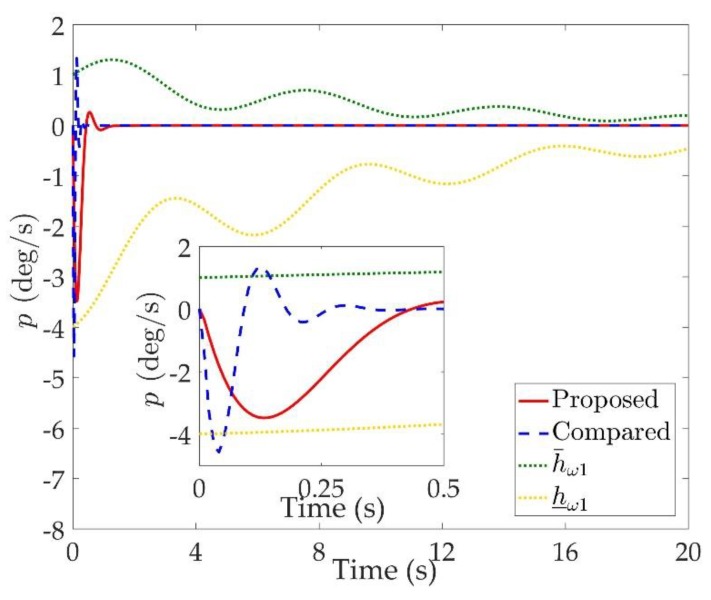
Comparison curves of the roll rate p.

**Figure 6:**
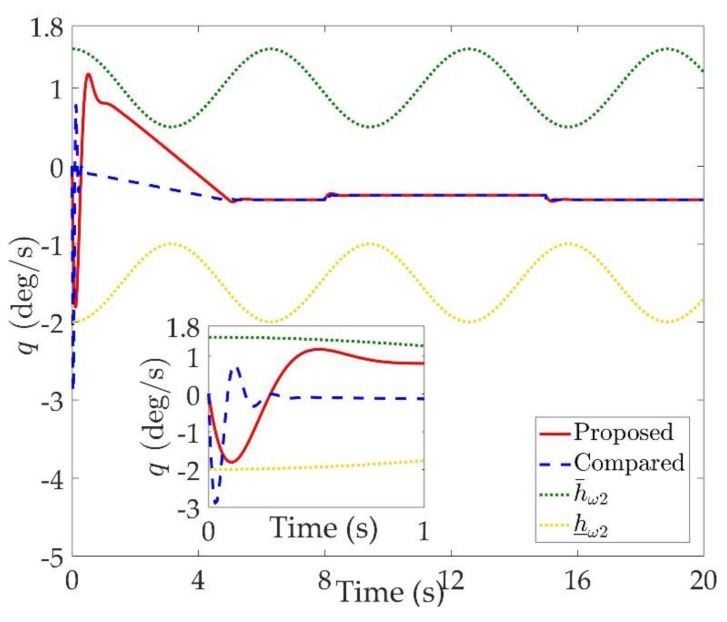
Comparison curves of the pitch rate q.

**Figure 7:**
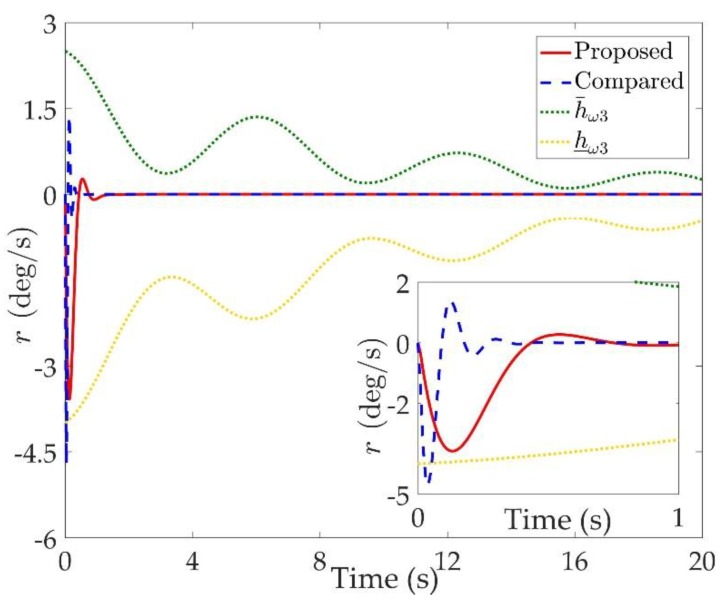
Comparison curves of the yaw rate r.

**Figure 8:**
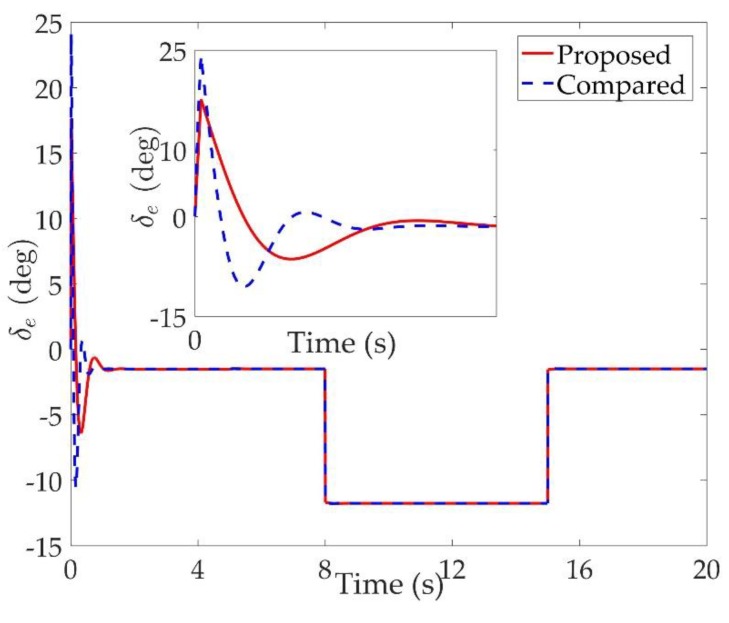
Comparison curves of the left elevon.

**Figure 9:**
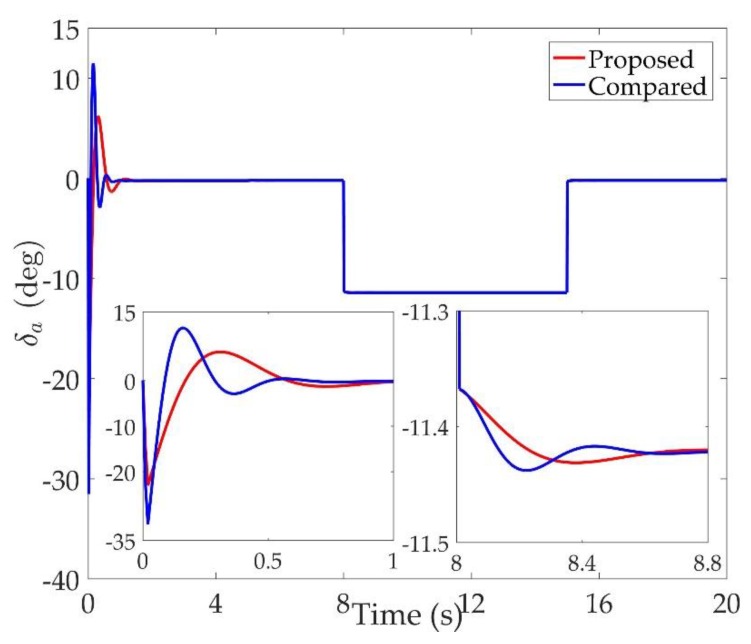
Comparison curves of the right elevon.

**Figure 10:**
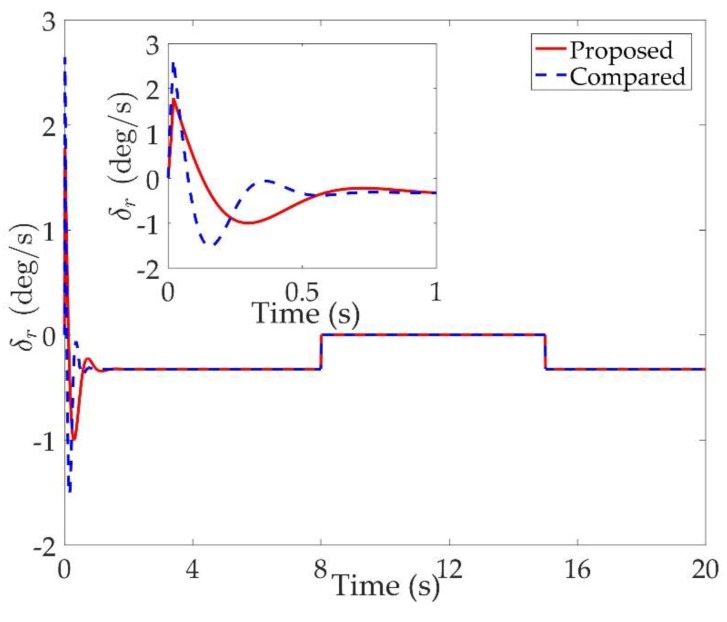
Comparison curves of the rudder.

**Figure 11:**
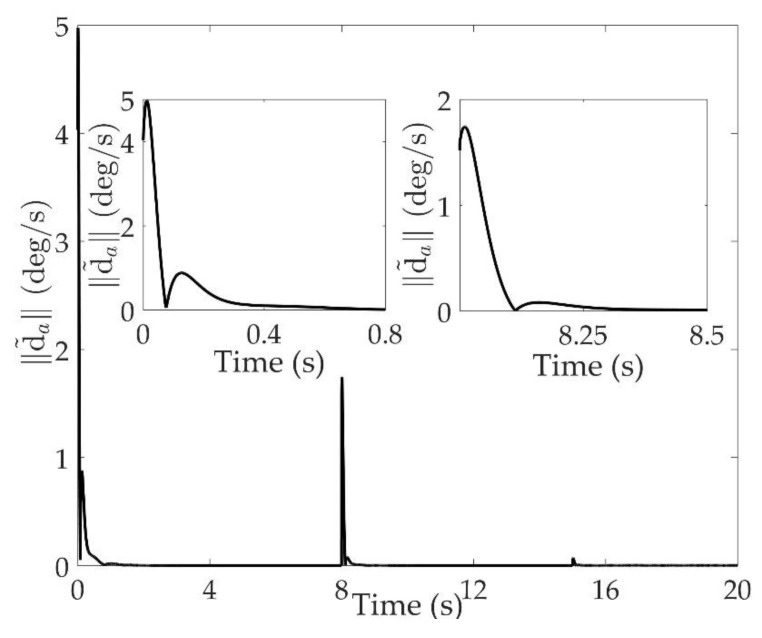
Estimation error of total disturbances $\left\|{\tilde{d}}_{a}\right\|$.

**Figure 12:**
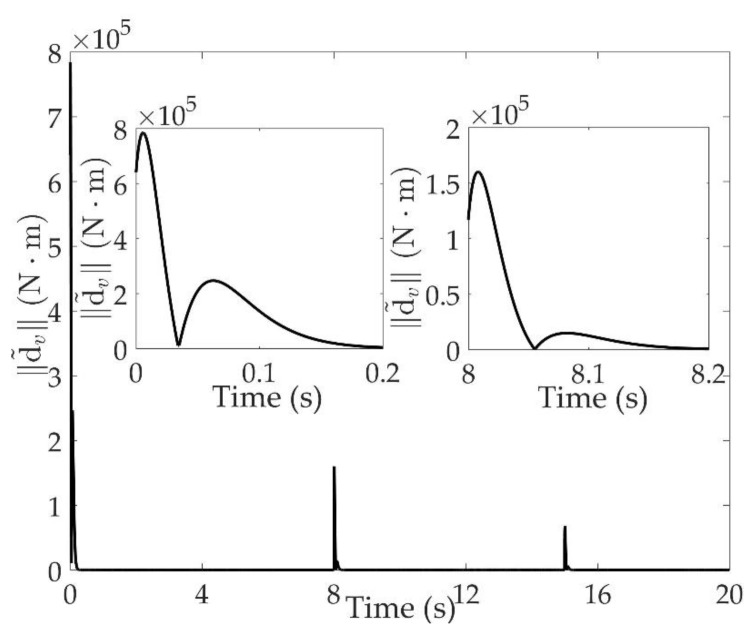
Estimation error of total disturbances $\left\|{\tilde{d}}_{v}\right\|$.

The simulation results are shown in Figure 2, Figure 3, Figure 4, Figure 5, Figure 6, Figure 7, Figure 8, Figure 9, Figure 10, Figure 11 and Figure 12. Figure 2, Figure 3 and Figure 4 are comparison curves of attitude angle tracking performance. Figure 5, Figure 6 and Figure 7 are comparison curves of angular rate performance. It can be seen that the desired signals are well tracked in the presence of aerodynamic coefficient uncertainties and external disturbances. The time-varying state constraints are not overstepped. Within a short time after the switching occurs, the tracking errors converge to a small residual set of zero. The attitude angle tracking errors are less than $0.05^{\circ }$ for t≥4 s. It should be pointed out that the angular rate responses in [10] exceed the state constraints at the beginning of the simulation, as shown in Figure 5, Figure 6 and Figure 7. With the help of the time-varying asymmetric BLF, the amplitudes of the state responses are all within a reasonable scope. Figure 8, Figure 9 and Figure 10 are comparison curves of control inputs. The control surface deflection angles in the proposed controller have smaller amplitudes and transition rates. It can be seen that the tracking curves of the proposed controller are slower than those compared, but the compared control law cannot guarantee that the states stay in the time-varying state constraints. To keep away from the state limit boundary as far as possible, the over control should be small—the tradeoff of this control scheme is the slow tracking speed. On the other hand, to guarantee the condition ${\underline{h}}_{\omega i}(t)<Z_{1i}+D_{\omega i}<{\bar{h}}_{\omega i}(t)$ as in the proof of Theorem 2, the control parameters should be chosen appropriately small. Although the proposed control method tracks the desired signal a little slower, the controller can guarantee the time-varying state constraints satisfied theoretically instead of adjusting parameters repeatedly.

The good tracking performance is on the basis of effective estimation of total disturbance. As shown in Figure 11 and Figure 12, the norm of estimation error ${\tilde{d}}_{a}$ is within 0.01 deg/s when 2 s<t<8 s, and within 0.01 deg/s two seconds after the system switching at 8 s and 15 s. The norm of estimation error ${\tilde{d}}_{v}$ is within 40 N⋅m when 2 s<t<8 s, and not exceeding 50 N⋅m one second after the switchings occur. Although the total disturbance is composed of modeling uncertainties and external disturbances, it can be seen that the proposed extended state observe can estimate the total disturbance accurately.

To implement the proposed controller, a gyroscope should be set on the VSNSV in order to obtain the attitude angle and angular rate information. The control input we designed in this study is the control torque vector which is generated by control surfaces including the left elevon, right elevon and rudder. The sensor and actuators necessary for the controller are common on variable-structure near-space vehicles [47]. Therefore, the proposed controller is easy to implement and applicable to most of the variable-structure near-space vehicles.

## 7. Conclusions

In this study, a solution to the problem of attitude tracking control and simulations of VSNSVs with time-varying state constraints are addressed. A novel ESO with two distinct linear regions is designed to estimate total disturbances. Then, based on the estimated values, the asymmetric time-varying barrier Lyapunov function is introduced to prevent the transgression of the state constraints. The modified dynamic surface control approach is presented to eliminate the ‘explosion of complexity’ inherent in the backstepping method. Rigorous proof for the convergence of ESO and the stability of closed-loop system is achieved. The numerical simulation results show the effectiveness of the proposed control scheme for VSNSV attitude tracking in the presence of disturbances. Further research may focus on considering the actuator saturation, actuator dead-zone, fault-tolerant control and the transformation of wings as an auxiliary control, while dealing with the time-varying state constraints for VSNSVs at the same time.
